# Gsα^R201C^ and estrogen reveal different subsets of bone marrow adiponectin expressing osteogenic cells

**DOI:** 10.1038/s41413-022-00220-1

**Published:** 2022-07-19

**Authors:** Biagio Palmisano, Rossella Labella, Samantha Donsante, Cristina Remoli, Emanuela Spica, Ilenia Coletta, Giorgia Farinacci, Michele Dello Spedale Venti, Isabella Saggio, Marta Serafini, Pamela Gehron Robey, Alessandro Corsi, Mara Riminucci

**Affiliations:** 1 Department of Molecular Medicine, Sapienza University of Rome, Rome, 00161 Italy; 2 Tettamanti Research Center, Department of Pediatrics, University of Milano Bicocca/Fondazione MBBM, Monza, 20900 Italy; 3 Department of Biology and Biotechnology, Sapienza University of Rome, Rome, 00185 Italy; 4 Institute of Structural Biology and School of Biological Sciences Nanyang Technological University, 639798 Singapore, Singapore; 5 CNR Institute of Molecular Biology and Pathology, Piazzale Aldo Moro 5, Rome, 00185 Italy; 6 Skeletal Biology Section, National Institute of Dental and Craniofacial Research, National Institutes of Health, Department of Health and Human Services, Bethesda, MD 20892 USA

**Keywords:** Bone, Pathogenesis

## Abstract

The Gsα/cAMP signaling pathway mediates the effect of a variety of hormones and factors that regulate the homeostasis of the post-natal skeleton. Hence, the dysregulated activity of Gsα due to gain-of-function mutations (R201C/R201H) results in severe architectural and functional derangements of the entire bone/bone marrow organ. While the consequences of gain-of-function mutations of Gsα have been extensively investigated in osteoblasts and in bone marrow osteoprogenitor cells at various differentiation stages, their effect in adipogenically-committed bone marrow stromal cells has remained unaddressed. We generated a mouse model with expression of *Gs*α^*R201C*^ driven by the *Adiponectin* (*Adq*) promoter. *Adq*-*Gs*α^*R201C*^ mice developed a complex combination of metaphyseal, diaphyseal and cortical bone changes. In the metaphysis, *Gs*α^*R201C*^ caused an early phase of bone resorption followed by bone deposition. Metaphyseal bone formation was sustained by cells that were traced by *Adq-Cre* and eventually resulted in a high trabecular bone mass phenotype. In the diaphysis, *Gs*α^*R201C*^, in combination with estrogen, triggered the osteogenic activity of *Adq*-*Cre*-targeted perivascular bone marrow stromal cells leading to intramedullary bone formation. Finally, consistent with the previously unnoticed presence of *Adq*-*Cre*-marked pericytes in intraosseous blood vessels, *Gsα*^*R201C*^ caused the development of a lytic phenotype that affected both cortical (increased porosity) and trabecular (tunneling resorption) bone. These results provide the first evidence that the *Adq-*cell network in the skeleton not only regulates bone resorption but also contributes to bone formation, and that the Gs*α*/cAMP pathway is a major modulator of both functions.

## Introduction

The α subunit of the stimulatory G protein (Gsα) is a ubiquitously expressed protein encoded by the *GNAS* gene (*GNAS* complex locus; *GNAS*, OMIM *139320), that couples cell surface receptors to adenylyl cyclase to stimulate cAMP production after ligand/receptor binding.^1,2^ The Gsα/cAMP signaling pathway participates in homeostasis of the post-natal skeleton by acting as a downstream effector of hormones and factors, such as parathyroid hormone, prostaglandin E2 and β-adrenergic agonists, which regulate the differentiation and function of different local cell types.^3,4^ Indeed, mutations of *GNAS* that lead to a gain of function of the Gsα protein (Gsα^R201C/R201H^) cause fibrous dysplasia of bone (Polyostotic FD/MAS, OMIM#174800) a disorder characterized by dysregulated bone resorption and abnormal bone formation that severely compromise the architecture and function of post-natal bone and bone marrow.^5,6^ How Gsα modulates the activity of cells within the bone and marrow compartments is a complex and still largely unresolved issue. However, significant insights were provided by transgenic mice in which a gain of function mutation of Gsα was expressed in a ubiquitous and constitutive manner,^7^ or at specific differentiation stages of maturation within the osteogenic lineage.^8,9^ Overall, these models demonstrated that dysregulated activity of Gsα in differentiated osteoblasts stimulates bone formation but does not affect the bone marrow microenvironment.^8^ In contrast, the expression of the mutation in early progenitor cells residing within the bone marrow stroma leads to a dramatic derangement of bone and marrow architecture and function.^7,9^ These results are consistent with current knowledge of the biology and activity of bone marrow osteoprogenitor cells. However, the bone marrow stromal cell (BMSC) system is heterogeneous and comprises not only osteoprogenitor cells but also contains progenitors committed to the adipogenic lineage.^10,11^ In particular, a marrow-specific adipogenic progenitor cell subset that expresses *Adiponectin (Adipoq, Adq)* (marrow adipogenic lineage precursors, MALPs) has been identified in mice.^11^ This *Adq*-*Cre*-targeted (*Adq*-) marrow stromal cell was identified as a major player in bone homeostasis due to its role in generation of marrow adipocytes, regulation of bone resorption and support of the marrow vasculature.^12^

As another step towards the definition of the role of Gsα signaling within bone and bone marrow stroma, and of the cellular pathogenesis of FD, we investigated the effects of expression of *Gs*α^*R201C*^ in marrow adipogenic cells by using the *Adipoq* promoter. We first demonstrate that the *Adq*-marrow stromal cell network was associated not only with an adipogenic function but also with an osteogenic activity that was manifested in the trabecular bone of most bone segments and increased during physiological mouse growth. We then show that the expression of *Gs*α^*R201C*^ in bone marrow *Adq-*cells enhanced both bone resorption and bone formation and generated a complex combination of metaphyseal, diaphyseal and cortical phenotypes. In the metaphysis of lumbar vertebrae and long bones, *Gs*α^*R201C*^ caused an early phase of enhanced bone resorption followed by a phase of enhanced bone formation driven by *Adq*-bone-forming cells. In the diaphysis, *Gs*α^*R201C*^ in combination with estrogen triggered the osteogenic activity of *Adq*-marrow perivascular/stromal cells leading to the deposition of intramedullary/perivascular bone. Finally, consistent with the existence of a previously unrecognized *Adq-*pericyte compartment associated with intraosseous blood vessels, we also observed that the expression of *Gs*α^*R201C*^ led to the development of a FD-like lytic phenotype affecting both cortical (increased porosity) and trabecular (tunneling resorption) bone.

## Results

### Adq-cells are found in the marrow cavity, trabecular bone and cortical blood vessel wall

**Fig. 1:**
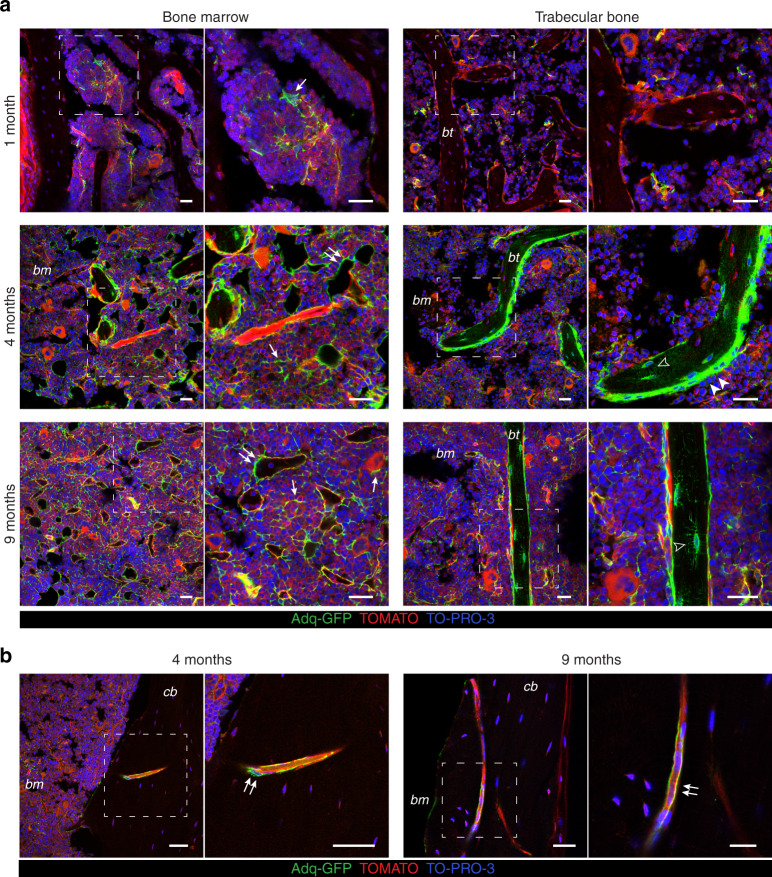
*Adipoq-Cre*-targeted cell population in bone marrow and bone. **a** Representative confocal images of bone marrow and trabecular bone from 1-, 4- and 9-month-old *Adq-mTmG* mice. GFP labeling was restricted to a small number of stromal cells at 1 month of age (*arrow*), while at 4 and 9 months it highlighted numerous marrow stromal (*arrow*) and perivascular (*double arrow*) cells, the majority of osteoblasts (*arrowhead*) and osteocytes (*hollow arrowhead*). **b** Representative fluorescent images of GFP-labelled intracortical perivascular cells (*double arrow*) in 4- and 9-month-old mice. *bm* Bone marrow, *bt* Bone trabecula, *cb* Cortical bone. Scale bars 25 μm

We assessed the distribution of *Adq*-cells in the mouse skeleton by crossing *Adq-Cre* mice with the *R26-mTmG* reporter line (Fig. S1a), such that cells remain red in the absence of *Adq-Cre* expression, and green when it is expressed. Confocal analysis of multiple skeletal segments from *Adq-Cre;R26-mTmG* (*Adq*-*mTmG*) mice showed different age- and site-dependent patterns of GFP labeling. In long bones from 1-month-old mice, GFP expression was observed in a subset of stromal cells within the marrow cavity (Fig. 1a) and among the trabeculae of the primary spongiosa (Fig. S1b). No labeling was detected in trabecular and cortical bone (Figs. 1a, S1b). However, at 4 and 9 months of age, the same skeletal segments showed extensive GFP expression in the marrow stroma among hematopoietic cells (Fig. 1a), where some of the GFP-positive cells were also stained by leptin receptor (LEPR) antibody (Fig. S1c). At 4 months, GFP-positive cells were also found in the primary spongiosa (Fig. S1b), around blood vessels (Figs. 1a, S1d), and also in many osteoblasts and osteocytes in trabecular bone (Figs. 1a, S1b). In contrast, only sparse osteocytes and some endosteal cells expressed GFP in the cortical bone (Fig. S1b). GFP expression in trabecular cells was observed in multiple skeletal segments except for the tail vertebrae and calvaria (Fig. S1e). In the tail vertebrae, the stromal compartment could not be visualized by confocal microscopy due to the massive presence of GFP-fluorescent marrow adipocytes, whereas in the calvaria only marrow stromal cells appeared to be labeled by GFP (Fig. S1e). Finally, as a previously unnoticed finding, we observed that throughout the skeleton and at all ages, some blood vessels running through the bony cortex also carried GFP-positive pericytes as observed in the marrow vasculature (Fig. 1b). Outside of the skeleton, GFP labeling was found in all fat depots but not in other tissues (Fig. S1f).

### Generation of transgenic mice expressing Gsα^R201C^ in Adq-cells

To assess the effect of Gsα overactivity on the biology and function of *Adq*-cells in bone, we crossed *Adq-Cre* mice with the *R26-lsl-Gs*α^*R201C*^ transgenic line expressing the R201C gain-of-function mutation of *Gs*α in a Cre-regulated manner (Fig. S2a). *Adq-Cre;R26-lsl*-*Gs*α^*R201C*^ (*Adq*-*Gs*α^*R201C*^) mice were viable and fertile. Consistent with the pattern of expression of GFP in *Adq-mTmG* mice, Cre recombination occurred in skeletal segments and in adipose depots but not in other tissues (Fig. S2b). The amount of the *Gs*α^*R201C*^ transcript within the skeleton of 3-month-old mice was comparable in the tail vertebrae and in bones with few to virtually no marrow adipocytes such as in calvaria, thus confirming the expression of the mutated Gsα sequence in all *Adq*-cells, and was particularly abundant in the tibia (Fig. S2c).

### The expression of Gsα^R201C^ in Adq-cells results in enhanced and unbalanced metaphyseal bone remodeling

**Fig. 2:**
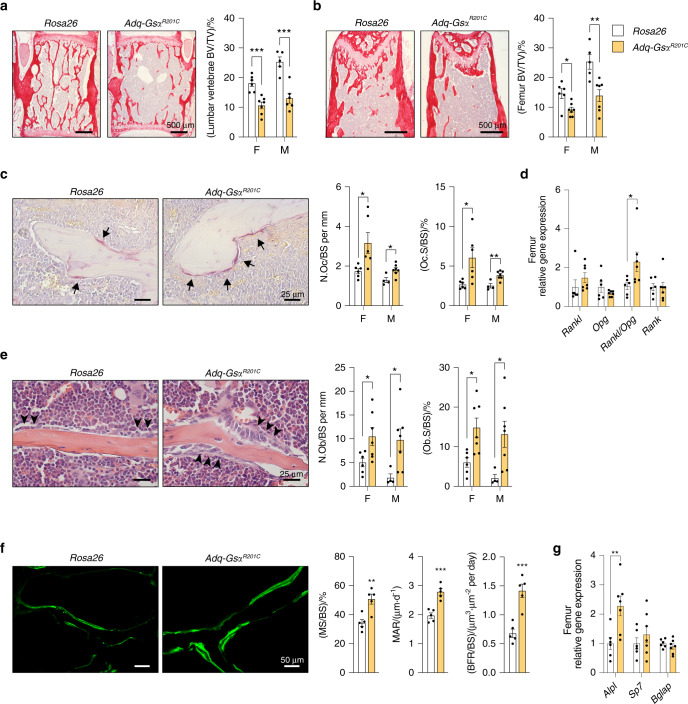
Enhanced trabecular bone resorption in 3-month-old *Adq*-*Gs*α^*R201C*^ mice. **a**, **b** Representative histological pictures from Sirius red-stained sections and histomorphometric analysis of trabecular bone of lumbar vertebrae (**a**) and femora (**b**). BV/TV: bone volume per tissue volume. **c** TRAP histochemistry highlighting cells of the osteoclastic lineage (*arrow*) and histomorphometry of osteoclast parameters. N.Oc/BS: number of osteoclasts per bone surface. Oc.S/BS: osteoclast surface per bone surface. **d** Relative gene expression of *Rankl*, *Opg, Rankl/Opg* ratio and *Rank* in femora from female mice. **e** Representative images from H&E-stained sections showing osteoblasts (*arrowhead*) and histomorphometry of osteoblast parameters. N.Ob/BS: number of osteoblasts per bone surface. Ob.S/BS: osteoblast surface per bone surface. **f** Representative pictures of calcein labeling in trabecular bone of female mice and dynamic histomorphometry for bone formation parameters. MS/BS: mineralizing surface per bone surface. MAR: mineral apposition rate. BFR/BS: bone formation rate. **g** Relative gene expression of *Alpl*, *Sp7* and *Bglap* in femora from female mice. F Females. M Males. Data are presented as mean ± SEM. Statistical analysis was performed using Student t-test; ^*^P < 0.05, ^**^P < 0.01, ^***^P < 0.001

**Fig. 3:**
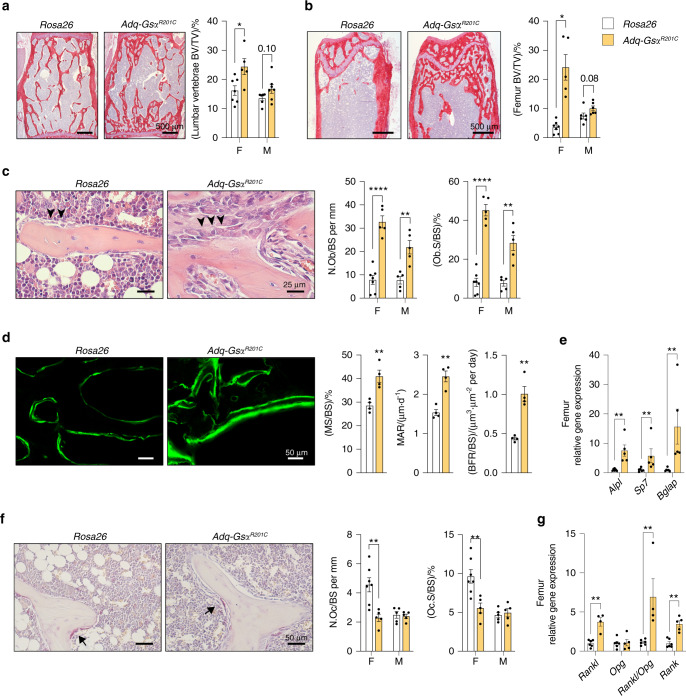
Enhanced metaphyseal bone formation in 9-month-old *Adq-Gs*α^*R201C*^ mice. **a**, **b** Representative histological pictures from Sirius red-stained sections and histomorphometric analysis of trabecular bone of lumbar vertebrae (**a**) and femora (**b**). BV/TV: bone volume per tissue volume. **c** Representative images from H&E-stained sections showing osteoblasts (*arrowhead*) and quantitative histomorphometry of osteoblast parameters. N.Ob/BS: number of osteoblasts per bone surface. Ob.S/BS: osteoblast surface per bone surface. **d** Representative pictures of calcein labeling in trabecular bone of female mice and dynamic bone formation parameters. MS/BS: mineralizing surface per bone surface. MAR Mineral apposition rate. BFR/BS Bone formation rate. **e** Relative gene expression of *Alpl*, *Sp7* and *Bglap* in femora from female mice. **f** TRAP histochemistry highlighting cells of the osteoclastic lineage (*arrow*) and quantitative histomorphometry of osteoclast parameters. N.Oc/BS: number of osteoclasts per bone surface. Oc.S/BS: osteoclast surface per bone surface. **g** Relative gene expression of *Rankl*, *Opg*, *Rankl/Opg* ratio and *Rank* in femora from female mice. F Females. M Males. Data are presented as the mean ± SEM. Statistical analysis was performed using Student *t*-test; ^*^P < 0.05, ^**^P < 0.01, ^****^P < 0.000 1. The exact *P*-value was reported on male column bars in **a** and **b**

The expression of *Gs*α^*R201C*^ in *Adq*-cells caused an enhanced and unbalanced trabecular bone remodeling that resulted in an opposite metaphyseal phenotype in young and old mice. At 3 months of age, both male and female *Adq*-*Gs*α^*R201C*^ mice showed osteopenic changes. In the lumbar vertebrae and distal femora, trabecular bone volume per tissue volume (BV/TV) was reduced (Fig. 2a, b) and histomorphometry on TRAP stained sections showed increased values of osteoclast number per bone surface (N.Oc/BS) and osteoclast surface per bone surface (Oc.S/BS) compared with *Rosa26* littermate controls (Fig. 2c). These findings associated with high values of *Rankl* transcript and *Rankl*/*Opg* transcript ratio in homogenized tissue samples (Fig. 2d). At the same time, histological and histomorphometric changes consistent with active osteoblast recruitment and function were also observed. Osteoblast number per bone surface (N.Ob/BS) and osteoblast surface per bone surface (Ob.S/BS) and dynamic parameters of bone formation were increased in *Adq-Gs*α^*R201C*^ mice (Fig. 2e, f). Moreover, the level of *Alpl* mRNA in homogenized bone tissue was higher compared with controls while no difference was found in the levels of *Sp7* and *Bglap* transcripts (Fig. 2g). Stimulation of osteogenic activity continued during mouse growth and in 9-month-old *Adq-Gs*α^*R201C*^ mice, the trabecular bone mass of the same skeletal segments was higher than in controls, especially in femora (Fig. 3a, b). At this age, bone surfaces were extensively rimmed by osteoblasts and the difference in the N.Ob/BS and Ob.S/BS values between *Adq-Gs*α^R201C^ and control mice was greater than that observed at 3 months, especially in females (Fig. 3c). Calcein labeling showed a significant increase in osteoblast activity parameters (Fig. 3d) and *Alpl*, *Sp7* and *Bglap* transcripts were also highly expressed in homogenized bone tissue (Fig. 3e). The osteoclast parameters normalized on trabecular bone surfaces were lower than controls in female *Adq-Gs*α^*R201C*^ mice (Fig. 3f) although the total number of osteoclasts per tissue area (N.Oc/TA: 15.22 ± 1.924 vs. 28.54 ± 4.064, Mean ± SEM, *P* < 0.01) and the level of *Rankl* expression (Fig. 3g) were still higher compared with controls. No significant difference was detected in the osteoclast parameters in male mice (Fig. 3f). Interestingly, bone marrow adiposity in long bones of *Adq-Gs*α^*R201C*^ mice was reduced at all ages, with marked differences observed in the number and area of bone marrow adipocytes in female mice (Fig. S3a, b).

### The bone Adq-cell network comprises metaphyseal osteoprogenitor cells that are regulated by Gsα

**Fig. 4:**
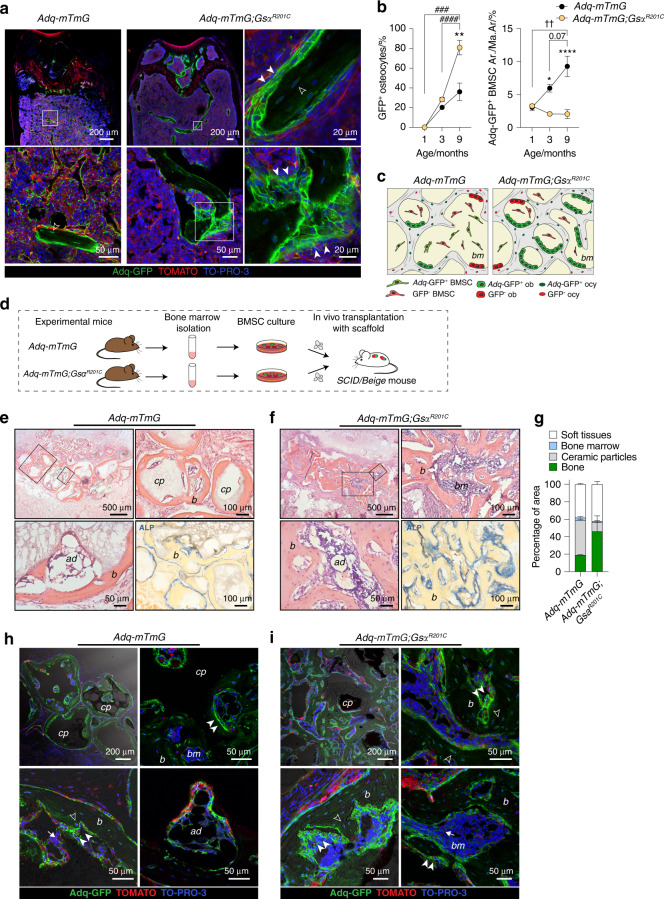
Osteogenic activity of *Adq-*marrow stromal cells in situ and in heterotopic transplants. **a** Representative images of trabecular bone from 9-month-old *Adq-mTmG and Adq-mTmG*;*Gs*α^*R201C*^ mice showing GFP-marked osteoblasts (*arrowhead*) and osteocytes (*hollow arrowhead*). **b** Quantification of the fraction of GFP-labeled osteocytes and the GFP-labeled stromal cells area in *Adq-mTmG and Adq-mTmG*;*Gs*α^*R201C*^ mice at different ages. Statistical analysis performed using Two-way ANOVA followed by Sidak’s multiple comparison test. ^*^P < 0.05, ^**^P < 0.01, ^****^P < 0.000 1 for comparison between *Adq-mTmG* and *Adq-mTmG*;*Gs*α^*R201C*^ mice. ^###^*P* < 0.001, ^####^*P* < 0.000 1 for comparison of different ages in *Adq-mTmG*;*Gs*α^*R201C*^ mice. ^††^*P* < 0.01, for comparison of different ages in *Adq-mTmG* mice. The exact *P*-value was reported for the comparison between 3 and 9 months in *Adq-mTmG* mice. **c** Scheme of changes in the trabecular bone mass and GFP-labeled osteoblasts, osteocytes and stromal cells in *Adq-mTmG* and *Adq-mTmG*;*Gs*α^*R201C*^ mice. **d** Experimental design for the heterotopic transplantation of BMSCs in SCID/beige mice. **e**, **f** H&E-stained section of transplants made with BMSCs derived from *Adq-mTmG* (**e**) and *Adq-mTmG;Gs*α^*R201C*^ (**f**) mice, showing newly generated bone (*b*), bone marrow (*bm*) and adipocytes (*ad*). Carrier particles (*cp*) were easily recognized only in *Adq-mTmG* samples. Numerous ALP positive stromal cells were detected in transplants generated with cells from *Adq-mTmG;Gs*α^*R201C*^ mice. **g** Quantification of fraction of transplant area occupied by soft tissue, bone marrow, ceramic particles and bone. **h**, **i** Representative confocal images of the same transplants showing GFP labeling in the majority of osteoblasts (*arrowhead*) and osteocytes (*hollow arrowhead*), in adipocytes (*ad*) and in stromal cells (*arrow*)

The trabecular osteopenic phenotype of 3-month-old *Adq*-*Gs*α^*R201C*^ mice was consistent with the previously reported stimulatory activity of *Adq*-stromal cells on bone resorption^12^ and revealed the regulatory role played by Gsα in this function. To assess the contribution of the same cells to the subsequent high metaphyseal trabecular bone mass phenotype we crossed *Adq-Gs*α^*R201C*^ mice with the *R26-mTmG* reporter line (Fig. S2d). Nine-month-old *Adq-mTmG*;*Gs*α^*R201C*^ mice showed a less dramatic increase in metaphyseal bone compared with age-matched *Adq-Gs*α^*R201C*^ mice, likely due to the presence of only one *R26-lsl*-*Gs*α^*R201C*^ allele. Nonetheless, confocal analysis of lumbar vertebrae and femora revealed the expression of GFP in the majority of osteoblasts and osteocytes in the metaphyseal bone trabeculae formed after the early resorption phase and an increased density of GFP positive stromal cells around their surface (Fig. 4a). The fraction of GFP-positive osteocytes increased over time in both *Adq*-*mTmG* and *Adq-mTmG;Gs*α^*R201C*^ mice, but at 9 months of age it was significantly higher in *Adq-mTmG;Gs*α^*R201C*^ mice compared with controls (Fig. 4b). In the bone marrow of *Adq*-*mTmG* mice, this was associated with an expansion of *Adq-*GFP marrow stromal area (Figs. 1a, 4b) that was not appreciated in *Adq-mTmG;Gs*α^*R201C*^ mice (Fig. 4a, b). These results suggest an active recruitment of *Adq-*GFP stromal cells to osteogenesis (Fig. 4c) and a role for Gsα in the stimulation of this process. To further support our findings, we performed heterotopic transplants with a mineralized osteoconductive carrier. BMSCs were isolated from long bones of 2-month-old *Adq-mTmG;Gs*α^*R201C*^ and *Adq*-*mTmG* control mice, loaded onto ceramic particles and transplanted into the back of immunocompromised mice (Fig. 4d). After 8 weeks, the control transplants showed abundant residual ceramic particles, the surfaces of which were extensively covered by lamellar bone, and marrow spaces occupied by hematopoietic cells and adipocytes (Figs. 4e, S4a). In contrast, the *Adq-mTmG;Gs*α^*R201C*^ transplants showed very few residual ceramic particles and a remarkable amount of haphazardly distributed bone (Fig. 4f, g). The newly formed bone had a mixed woven and lamellar structure (Fig. S4a), and its surfaces were covered by numerous TRAP positive osteoclasts (Fig. S4b). In these samples, the marrow spaces contained focal clusters of hematopoietic cells and adipocytes and were largely filled with ALP positive stromal cells (Fig. 4f). Confocal analysis demonstrated GFP-expressing osteoblasts and osteocytes in control transplants (Fig. 4h), thus supporting the existence of an *Adq*-stromal cell population in mouse long bone that is inherently osteogenic. In parallel with the higher amount of bone, GFP-labeled cells were numerous in *Adq-mTmG;Gs*α^*R201C*^ transplants in which GFP expression was also found within the ALP positive marrow stroma (Fig. 4i).

### The marrow Adq-cell network comprises diaphyseal osteoprogenitor cells that are regulated by Gsα and estrogen

**Fig. 5:**
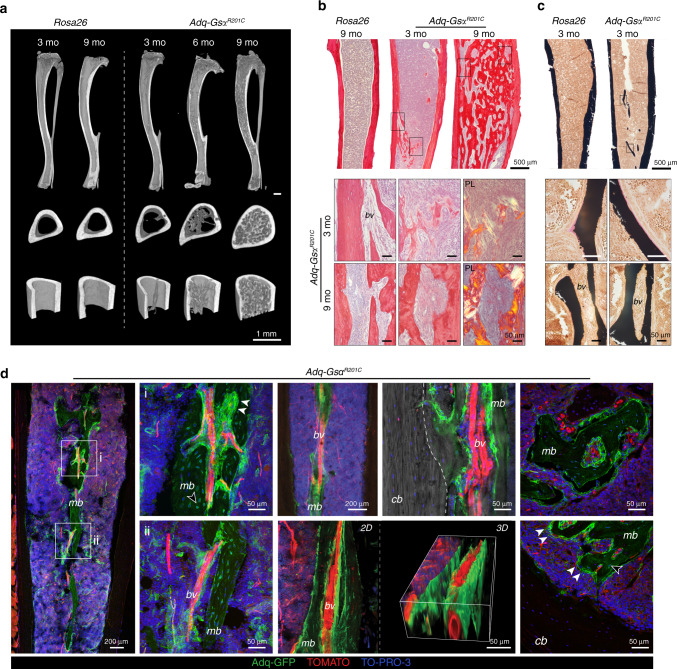
Diaphyseal intramedullary bone formation in female *Adq-Gs*α^*R201C*^ mice. **a** Representative micro-computed tomography images of mice at different ages, showing the appearance and progression of the diaphyseal intramedullary bone. Transverse images were taken 2 mm above the tibia-fibular junction. **b** Transmitted and polarized (PL) light microscopy views of Sirius red stained sections of the tibial midshafts showing the appearance of the intramedullary bone in *Adq-Gs*α^*R201C*^ mice at 3 months of age and its subsequent expansion with obliteration of the medullary canal at older ages. PL shows the mixed woven and lamellar bone structure. **c** Von Kossa/Van Gieson stained MMA sections of undecalcified tibial midshafts showing mineralized intramedullary bone with a thin layer of osteoid rimmed with osteoblasts. **d** Representative images from confocal microscopy, showing medullary bone (*mb*) distributed around marrow blood vessels (*bv*) and its focal connection with cortical bone (*cb, dotted line*). GFP expression was observed in osteoblasts (*arrowhead*) and osteocytes (*hollow arrowhead*). 3D reconstruction was performed on a 50 μm-thick section using ImageJ software

In addition to the metaphyseal bone changes, *Adq*-*Gs*α^*R201C*^ mice also developed an unexpected skeletal phenotype characterized by bone formation within the diaphyseal marrow cavity. The medullary osteogenic process was restricted to females and to long bones, with the tibia being the first involved segment in all female mice (Fig. S5a). Bone deposition started within 3 months of age in the diaphyseal region at the tibia/fibula junction, and, as clearly shown by micro-CT scans, it involved the cortical endosteum only focally (Fig. 5a), especially at sites in which large blood vessels entered the marrow cavity (Fig. 5b). Bone formation continued along the blood vessel wall to extensively fill the marrow space in old animals (Figs. 5a, b, S5a-c). The bone tissue had a mixed woven and lamellar structure (Fig. 5b), showed a thin layer of osteoid, and was well mineralized (Fig. 5c). In *Adq-mTmG;Gs*α^*R201C*^ female reporter mice the expression of GFP was detected in the vast majority of osteoblasts and osteocytes of the bone tissue wrapping the marrow vessels and in a few osteocytes in the inner side of the cortex in continuity with the medullary bone (Fig. 5d).

**Fig. 6:**
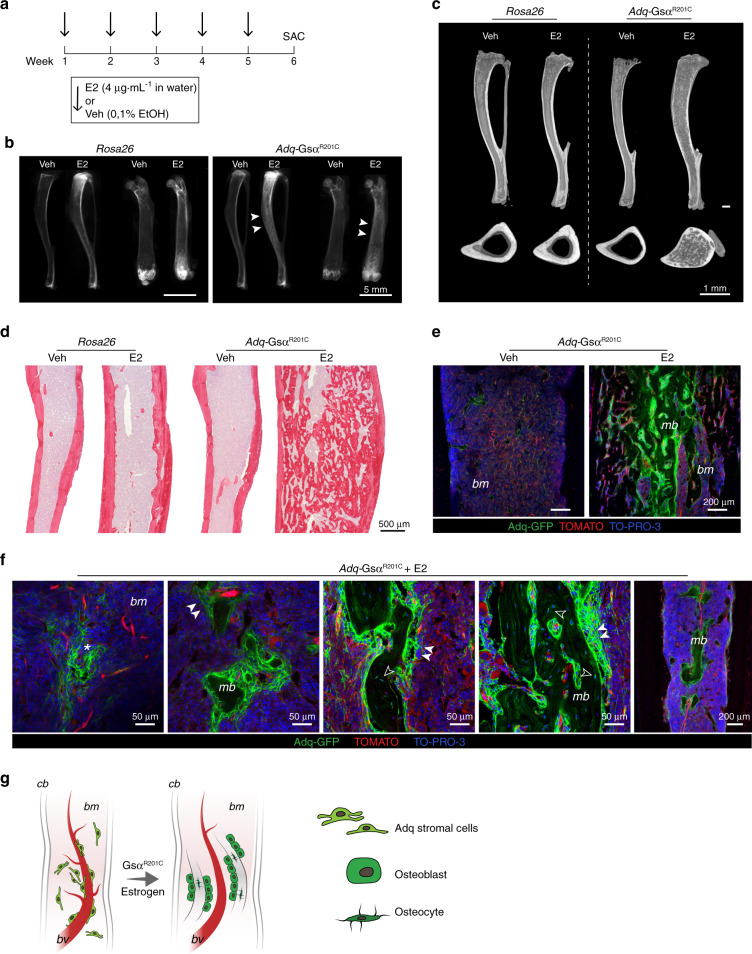
Diaphyseal intramedullary bone formation is reproduced in *Adq-Gsα*^*R201C*^ male mice by 17β-estradiol (E2) treatment. **a** Experimental scheme of E2 treatment started at 5 months of age. **b** Radiographs of dissected tibiae and femora at the end of E2 treatment. Arrowheads indicate the increased density in the diaphyseal region of E2-treated bone segments from *Adq-Gsα*^*R201C*^ mice. **c** Representative longitudinal and transverse micro-CT images of Veh- and E2-treated mice, showing the diaphyseal intramedullary bone in E2-treated *Adq-Gsα*^*R201C*^ male mice. Transversal images were taken 2 mm above the tibia-fibular junction. **d** Sirius red stained sections of the tibial midshafts showing intramedullary bone in *Adq-Gsα*^*R201C*^ male mice after 6 weeks of E2 treatment. **e** Representative confocal images showing GFP-labeled intramedullary bone in E2-treated *Adq-Gsα*^*R201C*^ male mice. No bone is observed in Veh-treated mice. **f** Cluster of perivascular GFP-labeled stromal cells (*asterisk*) preceding the appearance of bone with GFP-labeled osteoblasts (*arrowhead*) and osteocytes (*hollow arrowhead*) in the marrow cavity of E2-treated *Adq-Gsα*^*R201C*^ mice. **g** Schematic representation of GFP-labeled medullary bone formation by *Adq-Gsα*^*R201C*^ marrow perivascular/stromal cells. *mb* Medullary bone, *bm* Bone marrow, *cb* Cortical bone, *bv* Blood vessel

The absence of medullary bone formation in *Adq*-*Gs*α^*R201C*^ male mice suggested that in the diaphysis of long bones, there is a subset of stromal/perivascular *Adq*-cells that are osteogenic and are modulated by Gsα but require estrogen stimulation to turn into bone-forming cells. To test this hypothesis, we treated 5-month-old *Adq*-*Gs*α^*R201C*^ and control male mice with either 17β-estradiol (E2) or vehicle (Veh) in the drinking water for 6 weeks (Fig. 6a). In mice expressing the Gsα mutation, E2 treatment led to deposition of bone in the diaphyseal marrow cavity (Fig. 6b–d) similarly to what was observed in untreated female mice bearing the same genotype (Fig. 5). The origin of the intramedullary bone was explored in the tibiae and femora of E2-treated *Adq-mTmG*;*Gs*α^*R201C*^ male reporter mice, in which it included only GFP positive osteoblasts and osteocytes (Fig. 6e–g). After E2 treatment, an increase in the radiodensity of the metaphysis of long bones was detected in all male mice independent of their genotype (Fig. 6b, c). Histomorphometric analyses of trabecular bone demonstrated that BV/TV (Fig S6a) and osteoblast parameters **(**Fig S6b) were significantly higher in *Adq-Gs*α^*R201C*^ mice upon E2 treatment compared with Veh-treated *Adq-Gs*α^*R201C*^ and E2-treated control mice, in the absence of significant changes in osteoclast parameters (Fig S6c).

### Osteogenic Adq-cells are not found in all skeletal segments

**Fig. 7:**
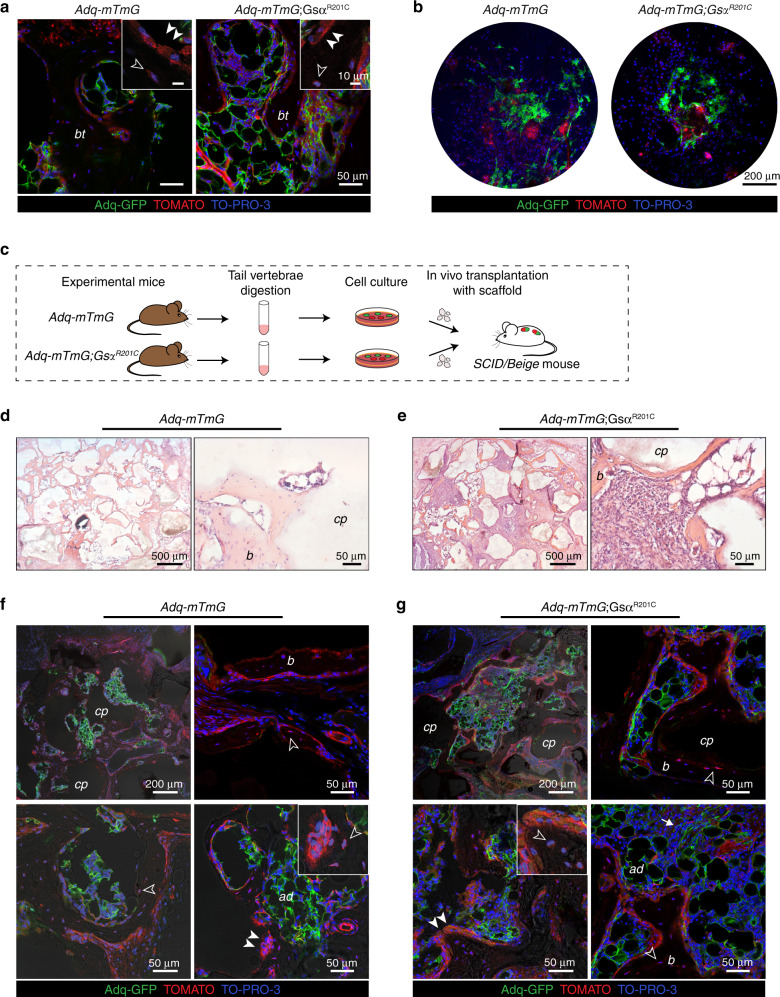
*Adq*-marrow stromal cells from tail vertebrae are not osteogenic. **a**, Representative confocal microscopy images of bone trabeculae (*bt*) in the tail vertebrae of *Adq-mTmG* and *Adq-mTmG*;*Gsα*^*R201C*^ mice showing only Tomato positive osteoblasts (*arrowhead*) and osteocytes (*hollow arrowhead*). **b** GFP-labeled adherent cells in BMSC cultures isolated from tail vertebrae of 2-month-old mice. **c** Experimental design for the heterotopic transplantation of bone marrow stromal cells isolated from tail vertebrae. **d**, **e** Representative transmitted light microscopy images of *Adq-mTmG* and *Adq-mTmG;Gsα*^*R201C*^ transplants showing newly formed bone on the surfaces of carrier particles and inter-particle spaces occupied by bone marrow and adipocytes. **f**, **g** Representative confocal microscopy images of the same transplants showing GFP labeling in adipocytes (*ad*) and stromal cells (*arrow*) within the inter-particle spaces. Only Tomato positive osteoblasts (*arrowhead*) and osteocytes (*hollow arrowhead*) were found in these transplants. *b* Bone, *cp* Carrier particles

As observed in control *Adq-mTmG* mice, the expression of GFP in trabecular osteoblasts and osteocytes was never detected in the tail vertebrae of *Adq*-*mTmG*;*Gs*α^*R201C*^ mice (Fig. S1e, Fig. 7a). This indicated the lack of osteogenic potential of *Adq*-cells in the tail. To further confirm this finding, and to rule out potential inhibitory effects of the local microenvironment, we performed heterotopic transplantation of adherent cells grown after enzymatic digestion of tail vertebrae. The cells were harvested from 2-month-old *Adq*-*mTmG*;*Gs*α^*R201C*^ and *Adq-mTmG* control mice, cultured in vitro (Fig. 7b) and transplanted with ceramic particles into the back of immunocompromised mice (Fig. 7c). After 8 weeks, bone tissue was detected in all samples (Fig. 7d, e). However, despite the presence of GFP-labeled adherent cells in the cell cultures (Fig. 7b) only Tomato positive osteocytes were detected by confocal analysis in all transplants (Fig. 7f, g). GFP labeling was restricted to marrow adipocytes (Fig. 7f, g) and, in *Adq*-*mTmG*;*Gs*α^*R201C*^ samples, to a small number of stromal cells distributed within the marrow spaces (Fig. 7g).

### The expression of Gsα^R201C^ in Adq-cells is associated with cortical lysis and trabecular tunneling (dissecting) resorption

**Fig. 8:**
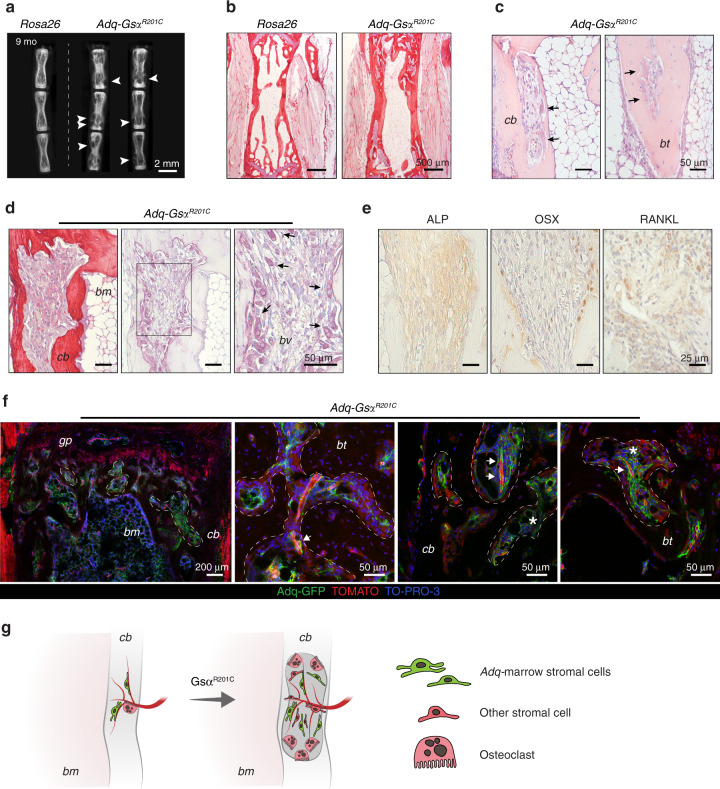
*Adq*-intraosseous pericytes associate with lytic lesions in cortical and trabecular bone in *Adq*-*Gsα*^*R201C*^ mice. **a** Radiographic analysis of dissected tail vertebrae from 9-month-old *Rosa26* and *Adq-Gs*α^*R201C*^ mice showing several osteolytic lesions (*arrowhead*). **b** Representative Sirius red stained sections of tail vertebrae from 9-month-old mice. **c** H&E-stained sections showing dissecting and tunneling resorption in tail vertebrae. Note the massive presence of osteoclasts (*black arrow*). **d** Sirius red and TRAP-stained sections showing intracortical bone resorption in tail vertebrae. Resorption areas progressed over time and were enriched in osteoclasts (*black arrow*) in close association with blood vessels (*bv*). **e** Immunolocalization of ALP, OSX and RANKL in a tail vertebra lytic lesion of *Adq*-*Gsα*^*R201C*^ mice. **f** Representative confocal images from *Adq-mTmG;Gsα*^*R201C*^ mice tail vertebrae showing areas of osteolysis (*dashed line*) filled with GFP-positive perivascular cells (*arrow*) and stromal cells (*asterisk*). **g** Schematic representation of lytic lesions associated with intracortical Gsα-mutated *Adq*-pericytes. *cb*: cortical bone, *bt*: bone trabecula, *bm:* bone marrow, *gp*: growth plate

Stimulation of bone resorption was a persistent feature of *Adq*-*Gs*α^*R201C*^ mice at all ages. While in young mice it involved predominantly the bone surface in the trabecular bone (Fig. 2a, b), during mouse growth, abnormal bone resorption was also observed around some blood vessels running within the cortical bone and in the larger, predominantly sub-cortical, bone trabeculae. Consequently, areas of lysis (increased porosity) and tunneling (dissecting) resorption developed in cortical and trabecular bone respectively. This phenotype was markedly expressed in the tail vertebrae (Fig. 8a–e) in which osteoclast-enriched lytic lesions (Fig. 8c, d) tended to expand over time and were always filled by a stromal tissue that was ALP-positive/OSX-negative and expressed RANKL, as assessed by immunohistochemistry (Fig. 8e). In *Adq*-*mTmG*;*Gs*α^*R201C*^ mice, GFP labeling was observed in perivascular cells and in some stromal cells within lytic regions, but not in the surrounding bone cells (Fig. 8f, g). Areas of cortical and trabecular bone resorption were also observed in other skeletal segments such as long bones and lumbar vertebrae (Fig. S7a). However, at these sites, the pattern of evolution was different compared with the tail since the resorption spaces were progressively replaced by hematopoietic marrow (Fig. S7b). In *Adq*-*mTmG*;*Gs*α^*R201C*^ mice, GFP labeling was localized to perivascular cells at sites of lesion development and in osteoblasts bordering the lytic area (Fig. S7c, d).

## Discussion

*Adipoq-Cre* recombination in the mouse skeleton occurs in marrow adipocytes, in a network of stromal cells residing among hematopoietic cells and around blood vessels, and in cells lining the trabecular bone surfaces.^11,13–15^ *Adq*-marrow stromal cells were reported to coincide with a fraction of *Lepr-Cre-*targeted stromal cells^13^ and to largely overlap with the PDGFRβ^+^/VCAM-1^+^/CXCL12^+^ (CAR) stromal cell subsets.^14^ In addition, it was shown that the majority of *Adq*-marrow stromal/perivascular cells are post-natal adipocyte progenitors, named MALPs, that support marrow vasculature and are involved in the regulation of bone resorption.^11,12^ Here we show that *Adq-Cre* in bone marrow targets a population that includes stromal cells, some of which express LEPR, and perivascular cells. This *Adq-Cre*-targeted cell population is associated with an osteogenic activity that is expressed in the metaphysis of different skeletal segments in physiological conditions and in the diaphysis of long bones under specific stimulatory settings.

*Adq-Cre* targeting of osteoblasts was previously noted by Zhou et al. as a rare event in vivo^13^ and by Mukohira et al. as an age-dependent phenomenon^14^ but neither further investigated its significance. In agreement with Mukohira’s report, we observed that in *Adq-mTmG* mice, GFP-labeled osteoblasts were virtually absent at a very young age, but their number progressively increased in the metaphyseal region of the long bones and lumbar vertebrae during mouse growth. This suggested the existence of *Adq*-marrow stromal osteoprogenitor cells that undergo progressive, age-dependent recruitment to trabecular bone formation and remodeling. This hypothesis was supported by the presence of GFP-positive osteoblasts and osteocytes in heterotopic ossicles made by BMSCs. Previously, other groups performed in vivo transplantation studies that failed to reveal the osteogenic potency of the *Adq*-marrow stromal compartment.^11^ Although we cannot exclude *Adipoq* promoter activation during the transplantation process, the expression of osteogenic activity in our transplants, could be ascribed to the different experimental conditions used in the in vivo assays compared with previous studies. For example, while in previous work, gelatin sponges (Gelfoam^TM^) were used as a carrier and the constructs were analyzed after 4 weeks,^11^ we transplanted the cells with a ceramic carrier and harvested the ossicles after 8 weeks. Thus, it is possible that the type of carrier and the longer time before harvesting in our experiments generated the optimal conditions for *Adq*-marrow stromal progenitors to express their osteogenic potency in vivo. In addition, we did not perform cell sorting before transplantation and we cannot exclude the possibility that *Adq*-cell survival and differentiation was favored in the presence of different populations of marrow stromal cells. Based on the evidence that *Adq*-marrow stromal cells represent an adipogenically committed cell population^11,12^ and that GFP-labeled adipocytes are also observed in our heterotopic ossicles, our data point to the existence of a subset of *Adq*-progenitors that are able to generate both adipocytes and osteoblasts.

The involvement of *Adq*-marrow stromal cells in both bone resorption and bone formation and the regulatory role played the Gsα/cAMP pathway in the two processes may explain the skeletal phenotype of *Adq*-*Gs*α^*R201C*^ mice. The Gsα/cAMP pathway is known to enhance the osteoclastogenic activity of marrow osteoprogenitor cells by initially stimulating the secretion of RANKL,^16–19^ which is physiologically produced by *Adq*-marrow stromal cells.^12^ On the other hand, Gsα is also known to stimulate cell commitment and subsequent bone formation within the osteogenic lineage.^8,20^ In *Adq*-*Gs*α^*R201C*^ mice at 3 months of age, increased *Rankl* expression by mutated stromal cells leads to the prevalence of trabecular bone resorption over bone formation, in spite of the increased osteoblastogenesis. During growth of the mouse, when the *Adq-*stromal compartment expands, as demonstrated in control *Adq-mTmG* mice, the Gsα mutation massively stimulates its osteogenic differentiation. Thus, in 9-month-old mice, bone formation prevails over bone resorption, leading to a high trabecular bone mass phenotype, despite the persistent increase in *Rankl* levels.

Of note, previous work demonstrated that in the bone marrow stromal compartment, Gsα enhances osteogenic differentiation at the expense of adipogenesis;^21,22^ accordingly, in *Adq*-*Gs*α^*R201C*^ mice, bone formation is associated with an overall reduction in bone marrow adipose tissue. We did not investigate the phenotypic effects and functional mechanisms affected by *Gs*α^*R201C*^ in *Adq*-marrow stromal cells. However, we showed that the events observed in *Adq*-*Gs*α^*R201C*^ mice were reproduced in heterotopic transplants in which the presence of *Adq*-*Gs*α^*R201C*^ marrow stromal cells associated with an unusual extensive “resorption”/disintegration of the ceramic particles^23^ and with the deposition of a high amount of bone that was largely made by GFP-positive bone cells.

Interestingly, our *Adq-Gs*α^*R201C*^ transgenic model also demonstrated that a synergy exists between Gsα^R201C^ and estrogen in stimulating bone formation by *Adq*-stromal cells within the diaphyseal marrow of long bones. The role of estrogen in this ectopic and non-structural osteogenic process was suggested by its spontaneous appearance in *Adq*-*Gs*α^*R201C*^ female mice and was confirmed by its reproduction in male mice treated with exogenous hormone. Estrogen-dependent bone deposition in the marrow diaphysis is a long-known phenomenon in birds, as a physiological calcium storage mechanism during the egg-laying cycle,^24^ and in female mice, as a non-physiological phenomenon induced by treatment with high doses of exogenous hormone.^25,26^ However, estrogen-related medullary osteogenic processes previously reported in birds and mice relied on the activation of cells lining the endosteal surface and resulted in a centripetal process of “endosteal trabecularization”.^25^ In our *Adq*-*Gs*α^*R201C*^ female mice and estrogen-treated male mice, the intra-medullary bone resulted from the activation of *Adq*-stromal cells on the blood vessel abluminal side and involved the cortical endosteum only focally, consistent with the discontinuous distribution of *Adq*-cells at this site. Thus, the topographic pattern of estrogen-dependent bone formation in the diaphyseal cavity of Gsα^R201C^ mice was rather reminiscent of that observed in some estrogen-independent experimental models of medullary osteogenesis, as for example, in colchicine treated rats.^27,28^ The reason why *Adq*-diaphyseal perivascular marrow stromal cells require both *Gs*α^*R201C*^ and estrogen to express their osteogenic potential remains to be addressed. However, it is interesting to note that recently, Sivaraj et al. demonstrated that diaphyseal stromal progenitors have different biological properties compared with their metaphyseal counterparts including a higher baseline commitment to adipogenesis.^29^ This finding may explain the different response of metaphyseal and diaphyseal *Adq*-marrow stromal cells to *Gs*α^*R201C*^ in our *Adq-Gs*α^*R201C*^ transgenic model. In metaphyseal *Adq*-marrow stromal cells, *Gs*α^*R201C*^ alone was sufficient to stimulate the osteogenic function that was further enhanced by estrogen. In contrast, in diaphyseal *Adq*-marrow stromal cells the synergic action of both stimuli was required to trigger and complete the osteogenic program.

Regardless of the mechanism of synergy of Gsα^R201C^and estrogen to enhance osteogenesis, the marrow osteogenic process in *Gs*α^*R201C*^ mice provides further in situ evidence of the ability of *Adq*-marrow stromal cells to form bone and has some interesting implications. First, it reveals that R201-mutated *Gs*α and estrogen together may induce an osteogenic fate choice in a subset of *Adq-*stromal cells that do not form bone in physiological conditions. Further investigation of this phenomenon may help to better understand the process of osteogenesis and to therapeutically approach low bone mass diseases. Second, consistent with Sivaraj’s work,^29^ it shows for the first time and in the intact bone, there is a major difference in the regulation of diaphyseal *Adq-Cre*-targeted osteoprogenitor cells compared with their metaphyseal counterparts and it seems to confirm the existence of an “internal plasticity”^29^ in the marrow stromal cell system. Finally, our data show that *Adq*-*Gs*α^*R201C*^ mice provide a unique model to study bone formation by marrow diaphyseal stromal cells in mammals in the absence of any type of mechanical or pharmacological manipulation of the local microenvironment and/or of hematopoiesis.

Interestingly, the metaphyseal and diaphyseal osteogenic activities of *Adq*-cells were not homogeneously distributed throughout the mouse skeleton since neither calvaria nor the tail vertebrae ever showed *Adq-Cre-* targeted osteoblasts and osteocytes. The reason for the lack of osteogenic activity of *Adq*-cells at these skeletal sites remains to be clarified. Meanwhile, we ruled out potential inhibitory effects of the local microenvironment by demonstrating that marrow stromal cells isolated from the tail of *Adq*-*mTmG;Gs*α^*R201C*^ mice did not form GFP-labeled bone cells in heterotopic transplants.

In this work, we also identified a compartment of *Adq*-perivascular cells within the cortical bone that was unnoticed in previous studies. Consistent with this finding and with the evidence that *Gs*α^*R201C*^ enhances the bone resorption stimulatory activity of *Adq*-marrow stromal cells, as shown in this study, *Adq-Gs*α^*R201C*^ mice developed a cortical lytic (increased porosity) and trabecular tunneling (dissecting) resorption phenotype that was particularly evident in the tail vertebrae. This phenotype may indicate a role for *Adq*-perivascular cells in the physiological remodeling of the transcortical vascular channels.^30^ In addition, it may shed some light on the cell type involved in the pathogenesis of Gsα-related skeletal diseases with abnormal bone resorption such as hyperparathyroidism and certain phases of lesion development in FD of bone.

FD is a severely crippling skeletal disorder associated with Gsα activating mutations.^5^ In FD lesions, normal bone is resorbed and replaced by fibrous marrow and newly formed, woven bone. We previously reported a FD phenotype in transgenic mice with ubiquitous expression of the *Gs*α^*R201C*^ mutation.^7^ Our current study shows that specific aspects of the FD phenotype are also reproduced in *Adq-Gs*α^*R201C*^ mice, thus revealing the involvement of the *Adq-*marrow stromal cell subset in the pathogenesis of the disease. Specifically, it suggests that Gsα-mutated *Adq-*marrow stromal cells might act as the trigger of the bone resorption processes (cortical lysis and dissecting resorption) that precedes the growth of the fibro-osseous tissue, and that they might also contribute to the deposition of FD bone. Interestingly, the post-natal appearance and progressive expansion of the *Adq*-marrow stromal cell network perfectly fit with the natural history of this skeletal dysplasia that, despite the early post-zygotic occurrence of the Gsα mutation, does not affect embryonic and fetal development of the skeleton, appears after birth, and worsens during skeletal growth.^31^ Thus, although *Adq-Gsα*^*R201C*^ mice fail to develop the full-blown FD skeletal phenotype observed in mice with ubiquitous and constitutive expression of *Gsα*^*R201C*^ ^7^, they could represent a useful tool to study the early stages of FD lesion development.

## Conclusion

In conclusion, in this work we demonstrated that *Adq-*cells in the marrow stromal cell network act not only as stimulators of bone resorption, but also as osteoprogenitor cells. We also showed that Gsα mediates their regulatory and progenitor functions and that the activity of the Gsα/cAMP signaling pathway must be tightly controlled in the entire *Adq*-marrow cell network in order to preserve the homeostasis of the post-natal bone/bone marrow organ.

## Materials and methods

### Mice generation

All studies were performed in compliance with relevant Italian laws and Institutional guidelines and all procedures were IACUC approved.

To generate the *Rosa26-lsl*-Gsα^R201C^ vector, the SalI/HindIII cassette was excised from the *R201C* rat *Gs*α cDNA, which included a hemagglutinin (HA) epitope as a flag (ATCC 63317, GenBank M12673),^32^ and inserted it into the pBigT vector (Addgene #21270), which contained a splicing acceptor site of adenovirus (SA), a stop region, including a neo cassette downstream to the PGK promoter and, downstream, a triple SV40 poly-adenylation sequence. The stop cassette was flanked by two *loxP* sites and followed by the bovine growth hormone poly-adenylation signal (BGHpA). The PacI and AscI restriction sites, placed 5’ to the SA and 3’ to the BGHpA, respectively, were used to subclone the functional sequence into the pRosa26PA plasmid (Addgene #21271), which included the *Rosa26* sequences required for targeted recombination into the murine *Rosa26* locus. *R26-lsl-Gs*α^*R201C*^ mice were generated by electroporating the *R26-lsl*-Gsα^R201C^ vector into murine ES CK35 (129/Sv Pas strain) cells. Upon electroporation, mouse ES cells were selected by neo screening, and 216 neo resistant clones were analyzed by multiple PCRs and sequencing. Two positive ES clones were implanted in C57Bl/6 N blastocysts, which, in turn were transferred into surrogate B6CBAF1 female mice. Mouse chimeras were backcrossed with C57Bl/6 N and F1 animals were genotyped by PCR. Heterozygous F1 mice were obtained from both founder clones. Two lines were serially backcrossed and showed regular Mendelian inheritance of the transgenic cassette.

Homozygous (homo) *R26-lsl-Gs*α^*R201C*^ mice (*Rosa26*) were crossed with heterozygous (het) *Adipoq-Cre* mice (#028020, The Jackson Laboratory); the resulting progeny were crossed again with Rosa26 mice to generate *Adiponectin-Cre*(het);*R26-lsl-Gs*α^*R201C*^*(*homo) (*Adq-Gs*α^*R201C*^) mice, which expressed the mutant form of *Gs*α in adipogenic cells.

To generate *Adq-mTmG* and *Adq-mTmG*;*Gs*α^*R201C*^ lineage reporter mice, we crossed heterozygous *Adq-Cre* and double heterozygous *Adq-Gs*α^*R201C*^ mice with homozygous *loxP-mT-pA-loxP-mG-pA* (*mTmG*) mice (#007676 The Jackson Laboratory). The triple heterozygous *Adq-mTmG;Gs*α^*R201C*^ mice harbor one R26 allele with *Gs*α^*R201C*^ transgene while the other *R26* allele contains the *mTmG* transgene.

All mice were maintained in cabin-type isolators at standard environmental conditions (temperature 22–25 °C, humidity 40%–70%) with 12:12 h dark/light photoperiod. Food and water were provided *ad libitum*. Mice were genotyped by using the oligonucleotides listed in Table 1.

**Table 1:** Sequence of primers used for genotyping and qPCR

**Genotyping**	
**Mouse strain**	**Sequence 5’ − 3'**
***Adipoq-Cre***	F: GCATTGCTGTCACTTGGTCGT
	R: CGATGCAACGAGTGATGAGG
***R26-lsl-Gsα***^***R201C***^	F: AAAGTCGCTCTGAGTTGTTAT
	R: GCGAAGAGTTTGTCCTCAACC
	R: GGAGCGGGAGAAATGGATATG
***R26-mTmG***	F: CTCTGCTGCCTCCTGGCTTCT
	R: CGAGGCGGATCACAAGCAATA
	R: TCAATGGGCGGGGGTCGTT
**qPCR**	
**Gene**	**Sequence 5’ − 3'**
*Alpl*	F: CCAGAAAGACACCTTGACTGTGG
	R: TCTTGTCCGTGTCGCTCACCAT
*Bglap*	F: GCAATAAGGTAGTGAACAGACTCC
	R: CCATAGATGCGTTTGTAGGCGG
*Col1a1*	F: CAGGGTATTGCTGGACAACG
	R: TTGTTTGCCAGGTTCACCAGA
*Gapdh*	F: CATCACTGCCACCCAGAAGACTG
	R: ATGCCAGTGAGCTTCCCGTTCAG
*HA (R26-Gnas)*	F: GAAGAGGACGTGCCGGATTAC
	R: TGGTTTCAATGGCCTCCTTCA
*Opg*	F: CGGAAACAGAGAAGCCACGCAA
	R: CTGTCCACCAAAACACTCAGCC
*Rank*	F: GGACAACGGAATCAGATGTGGTC
	R: CCACAGAGATGAAGAGGAGCAG
*Rankl*	F: CGAGCGCAGATGGATCCTAA
	R: GCAGGAGTCAGGTAGTGTGT
*Runx2*	F: CCTGAACTCTGCACCAAGTCCT
	R: TCATCTGGCTCAGATAGGAGGG
*Sp7*	F: GGCTTTTCTGCGGCAAGAGGTT
	R: CGCTGATGTTTGCTCAAGTGGTC

### X-ray analysis and micro-CT scanning

Radiographic analyses were performed on femora and tibiae using Faxitron MX-20 Specimen Radiography System (Faxitron X-ray Corp., Wheeling, IL, USA) set at 24–25 kV for 6–8 s with Kodak MIN-R2000 18 × 24 films.

For micro-CT scanning, tibiae were placed within a plastic tube, mounted onto the instrument rotational stage and scanned at 8 μm voxel size using 80 kV, 50 μA X-ray settings and a 1 mm aluminum filter with exposure time of 100 ms per frame, with a Bruker SkyScan 1275 micro-CT (Micro Photonic, Allentown, PA, USA). Three-dimensional reconstruction was performed with Bruker’s NRecon software and visualization occurred using Bruker’s DataViewer and CTscan software programs.

### Histology

Mice were euthanized by carbon dioxide inhalation and skeletal segments were dissected and processed for either paraffin embedding, methylmethacrylate (MMA) or gelatin embedding.

For paraffin embedding, samples were fixed with 4% formaldehyde in PBS pH 7.4 for 48 h at 4 °C and decalcified in 10% EDTA for 14–21 days at 4 °C with gentle shaking. Three-micron-thick sections were used for standard histology after staining with Hematoxylin-Eosin (H&E) or with Sirius red to visualize collagen fibers, for Tartrate-Resistant-Acid-Phosphatase (TRAP) histochemistry to highlight cells of the osteoclastic lineage and for histomorphometry.

MMA embedding was performed on undecalcified bone segments. After dissection, bone samples were fixed in 4% formaldehyde for 24 h and dehydrated through a series of increasing ethanol concentrations. Bones were then infiltrated for 3 days with the plastic embedding mixture containing 60 mL of MMA, 35 mL butylmethacrylate, 5 mL methylbenzoate, 1,2 mL polyethylene glycol 400 and 0.8 g of dry benzoyl peroxide. The polymerization mixture was prepared by adding 400 μL of N,N-dimethyl-p-toluidine to the infiltrating solution. Sections of 4–7 μm in thickness were cut from MMA blocks, deplasticized with 2-methoxyethylacetate (all reagents were purchased from Sigma Aldrich, Saint Louis, MO, USA), stained with Von Kossa and counterstained with Van Gieson.

For gelatin embedding, freshly dissected femora, tibiae and tail vertebrae were fixed in cold 4% formaldehyde solution for 4 h, washed in 1X PBS and decalcified in 0.5 M EDTA at 4 °C. Soft tissues were fixed in 4% formaldehyde for 4 h. Samples were then placed in 20% sucrose and 2% Polyvinylpyrrolidone (PVP) solution in PBS for a further 48 h. Samples were embedded in an 8% porcine gelatin solution containing 20% sucrose and 2% PVP as previously reported.^33^ Twenty to 50 μm-thick sections were cut, air-dried for 30 min, hydrated with 1X PBS, stained with TO-PRO-3 (#T3605, Thermo Fisher Scientific, Waltham, Massachusetts, USA) for nuclei visualization and imaged with Leica Confocal Microscope (Wetzlar, Germany). For heterotopic transplants, gelatin embedded samples were also used to perform TRAP and Alkaline Phosphatase (ALP) histochemistry.

For measurements of GFP-positive BMSC area (*Adq*-GFP^+^ BMSC Ar/Ma.Ar), pictures at 40X magnification were taken with Leica Confocal Microscope, color channel split by ImageJ software and green channel used for quantification of the area of signal that was then normalized on the marrow area. The fraction of GFP-labeled osteocytes (GFP^+^ osteocytes) was calculated by counting them on bone trabeculae of femora and tibiae.

### Histochemistry

TRAP and ALP histochemistry were performed using Sigma Aldrich reagents (Sigma Aldrich). Briefly, for TRAP histochemistry working solution, 50 mg of Naphtol AS-BI phosphate were dissolved in 4 mL N,N-dimethylformamide added to 4 mL acetate buffer and 92 mL of distilled water; then, 150 mg of Tartaric Acid and 30 mg of Fast Garnet were added. The slides were incubated with the working solution at 37 °C. For histochemical detection of ALP on gelatin sections, 30 mg of Naphtol AS Phosphate were dissolved in 0,5 mL N,N-dimethylformamide and added to a 100 mL borate buffer with 100 mg of AS blue BB salt. The solution was added to the slide and incubated for 5–10 min at 37 °C.

### Immunohistochemistry

Immunolocalization of OSX, RANKL and ALP was performed using rabbit anti-mouse antibodies (anti-OSX #ab22552, anti-RANKL #ab37415, Abcam, Cambridge, UK; anti-ALP # 11187–1-AP, Proteintech, Rosemont, Illinois, USA) applied at a dilution of 1:200 in PBS + 1% turn BSA, overnight at 4 °C. After repeated washing with PBS, sections were incubated for 30 min with biotin-conjugated swine anti-rabbit IgG (#P0217, Agilent, Santa Clara, CA, USA) 1:200 in PBS + 1% BSA and then exposed for 30 min to peroxidase-conjugated ExtrAvidin (#P0217, Agilent) (1:50 in PBS + 1% BSA). The peroxidase reaction was developed using DAB substrate kit (SK-4100, Vector Laboratories, Burlingame, CA, USA).

Immunolocalization of LEPR, endomucin (EMCN) and alpha-smooth muscle actin (α-SMA) was performed using goat anti-LEPR (#AF497, R&D Systems, Minneapolis, MN, USA), rat anti-EMCN (#ab106100, Abcam) and rabbit anti-α-SMA (#ab5694, Abcam). Twenty-five μm-thick sections from gelatin-embedded samples were rehydrated with PBS and then immunostained overnight. After primary antibody incubation, sections were repeatedly washed with PBS and incubated with appropriate Alexa Fluor 647-conjugated (rabbit anti-goat IgG #A-21446, goat anti-rat IgG #A-21247, goat anti-rabbit IgG #A27040 Thermo Fisher Scientific) secondary antibodies for 1 h at room temperature. Nuclei were counterstained with TO-PRO3.

### Histomorphometry

Quantitative bone histomorphometry was conducted on lumbar vertebrae (3^rd^ and 4^th^) and on distal femora. Experiments were performed in a blinded fashion. Different bone parameters, using standard nomenclature and abbreviations,^34^ were measured in a region of interest (ROI) in the secondary spongiosa of distal femora, starting 300 μm below the growth plate and for a length of 1 mm, and between the two growth plates in lumbar vertebrae. H&E and Sirius red-stained sections were used to measure trabecular bone volume per tissue volume (BV/TV), osteoblast number per bone surface (N.Ob/BS) and osteoblast surface per bone surface (Ob.S/BS). TRAP-stained sections were used to measure osteoclast number per bone surface (N.Oc/BS) and osteoclast surface per bone surface (Oc.S/BS).

Dynamic bone histomorphometry was performed on lumbar vertebrae dissected from mice that were treated with 30 mg/kg of calcein (Sigma Aldrich), 5 and 2 days before euthanasia. Calcein fluorescent labeling was used to quantify mineralizing surface (MS/BS), mineral apposition rate (MAR) and bone formation rate (BFR/BS).

Bone marrow adiposity was analyzed by manual counting of the number of adipocytes per marrow area (N.Ad/Ma.Ar) and by measuring their area (Ad.Ar/Ma.Ar) in H&E-stained sections of distal femora, according to standard procedures and nomenclature.^35,36^

Pictures were acquired with an optical microscope (Zeiss Axiophot, Jena, Germany) through a digital camera (Jenoptik ProgrRes C5, Jena, Germany) and all histomorphometric analyses were performed using ImageJ.^37^

### Transplantation assay

BMSCs were isolated from 3-month-old female *Adq-mTmG* and *Adq- mTmG;Gs*α^*R201C*^ mice by flushing long bones (femora, tibiae, humeri) and crushing sacral and lumbar vertebrae. Cell suspensions were collected after vigorous pipetting, filtered through a 70 μm nylon mesh cell strainer, and grown at 37 °C 5% CO_2_ as multiclonal cell strains^38^ in αMEM Sigma Aldrich) supplemented with 20% Fetal bovine serum (Thermo Fisher Scientific), 1% L-glutamine, 1% penicillin/streptomycin Sigma Aldrich). To isolate BMSCs from the tail vertebrae, the tails were skinned, cleaned of muscle and tendons and incubated in 5 mL of 2 mg·mL^−1^ Collagenase I solution in HBSS for 15 min and then spun at 200 *g* (relative centrifugal force) to remove the periosteum. The vertebrae were then minced and incubated in 10 mL of 2 mg·mL^−1^ Collagenase solution in HBSS for 1 h and spun at 100 *g*. After digestion, the supernatant was collected, filtered through 70 μm nylon mesh and centrifuged at 1 300 *g* for 6 min. The resulting pellet was washed with αMEM and cells were grown in the supplemented culture medium reported above.

After 1 week, all cell populations were transplanted. Constructs were made by loading 7 × 10^6^ cells onto 40 mg of ceramic particles (a component of AttraX, NuVasive, San Diego, CA, USA) and transplanted into 2-month-old female CB17.Cg-Prkd^scid^Lyst^bg-j^/Crl (SCID/beige) mice (Charles River, Wilmington, Massachusetts USA) as previously described.^39^ After 8 weeks, samples were harvested, fixed in 4% formaldehyde in PBS pH 7.4 for 12 h and decalcified in 0.5 mol·L^−1^ EDTA for 1 week. Samples were then processed for porcine gel embedding as described above. Ten-micron-thick sections were stained with H&E for morphology evaluation and histomorphometric quantification of the different tissue area, and for TRAP and ALP histochemistry. Twenty μm-thick sections were cut and analyzed by confocal microscopy for visualization of GFP and tdTomato fluorescence.

### Estradiol treatment

For 17β-estradiol (E2, Sigma Aldrich) treatment, two experimental groups were established: a vehicle group including *Rosa26*, *Adq*-*Gs*α^R201C^ and *Adq*-*mTmG*;*Gs*α^R201C^ mice (*n* ≥ 4 for each genotype, 5 months of age) and a E2 group including *Rosa26*, *Adq-Gsa*^R201C^ mice, *Adq*-*mTmG*;*Gs*α^R201C^ mice (*n* ≥ 4 for each genotype, 5 months of age). E2 was dissolved in ethanol at a concentration of 5 mg·mL^−1^ and added to 300 mL of drinking water to a final concentration of 4 μg·mL^−1^. The vehicle group received ethanol in drinking water at a final concentration of 0.1%. Water bottles were changed every week. The dose of E2 ingested was calculated as previously reported.^40^ After 6 weeks of treatment, mice were sacrificed by CO_2_ inhalation. Radiographic analyses were performed before the treatment and at sacrifice.

### Gene expression analysis by quantitative PCR

Femora and tibiae from 3- and 9-month-old female mice were dissected and snap frozen in liquid nitrogen and kept at −80° until use. Bone samples were homogenized by Mikro-Dismembrator U (Gottingen, Germany) and total RNA was isolated using the TRI Reagent® (Thermo Fisher Scientific) protocol. Reverse transcription was performed by using QuantiTect® Reverse Transcription Kit (Qiagen, Hilden, Germany). cDNA samples were used as templates for quantitative PCR (qPCR) analysis on a 7500 Fast Real-Time PCR System (Applied Biosystem, Waltham, Massachusetts, USA), performed using PowerUP Sybr Green (Thermo Fisher Scientific) and specific primers (Table 1). Gene expression levels of each gene were normalized to GAPDH expression.

### Statistical analysis

The comparisons between two groups were performed using the unpaired t-test. Changes in the GFP^+^ osteocyte fraction and *Adq-*GFP^+^ BMSC Area between *Adq-mTmG* and *Adq-mTmG;Gs*α^*R201C*^ mice at different ages were analyzed with the two-way ANOVA followed by a Sidak’s multiple comparison test. The comparison of histomorphometrical parameters during estrogen treatment was performed using one-way ANOVA followed by a Tukey’s multiple comparison test. In all experiments a *P*-value less than 0.05 was considered statistically significant. All graphs and statistical analyses were performed using GraphPad Prism version 8 (GraphPad Software, La Jolla, CA, USA).

## Supplementary information

Supplementary figure and legends

## Data Availability

The authors declare that all data supporting the findings of this study are available within the paper and its supplementary information files.
