# Supplementary material for: GsαR201C and estrogen reveal different subsets of bone marrow adiponectin expressing osteogenic cells
Source: Bone Res. 2022 Jul 19;10:50. doi: 10.1038/s41413-022-00220-1 (PMC9296668; doi:10.1038/s41413-022-00220-1)
Supplement: Supplementary file 1 — Supplementary figure and legends [file 41413_2022_220_MOESM1_ESM.docx]

**Figure S1.**

**
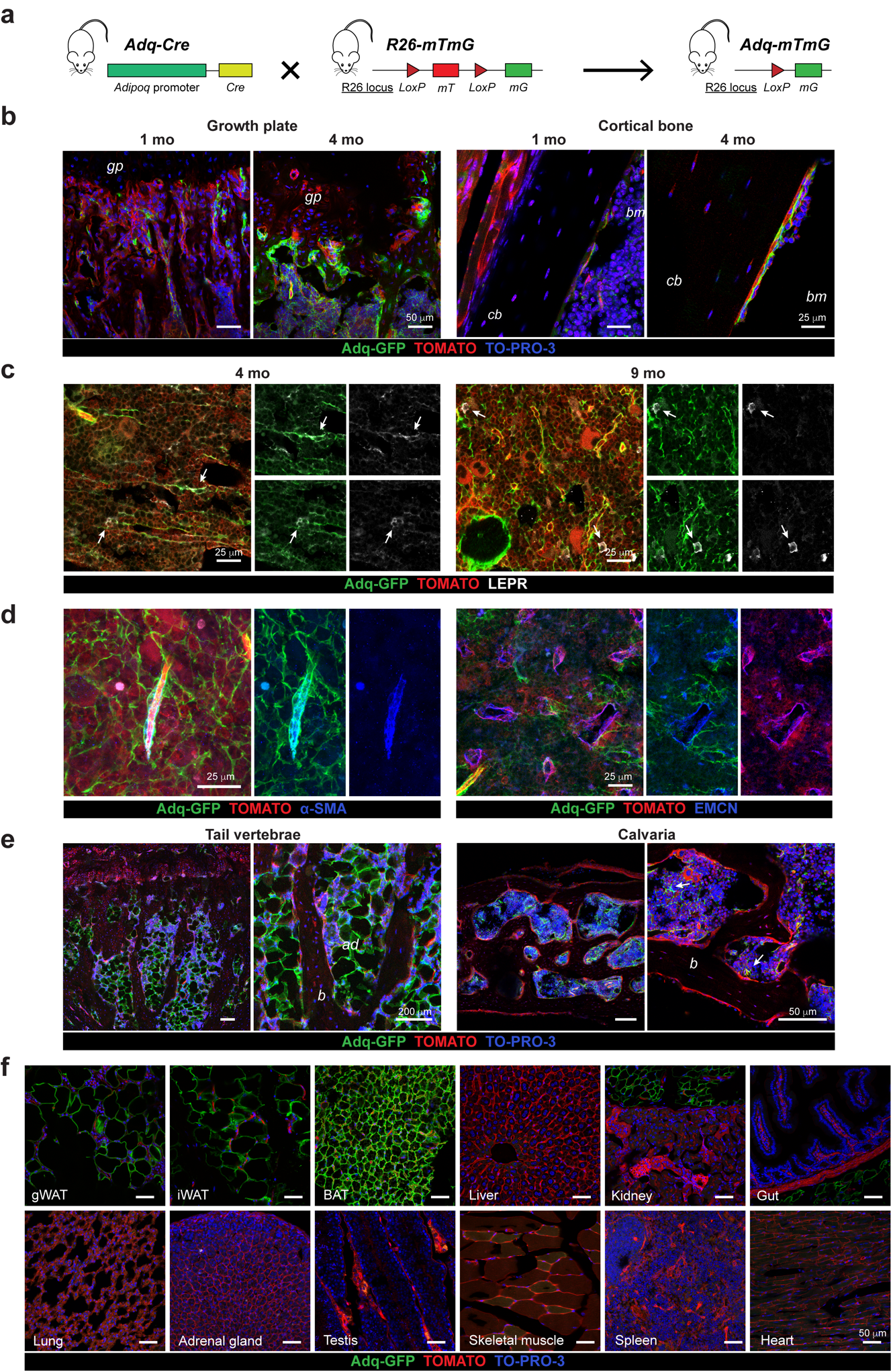
**

**Fig. S1 | a,** Experimental scheme showing the generation of *Adq-mTmG* mice. **b**, Representative confocal images of femur growth plate and cortical bone from 1- and 4-month-old *Adq-mTmG* mice. **c**, Confocal images of the femur bone marrow of a 4- and 9-month-old *Adq-mTmG* mice immunostained with leptin receptor (LEPR) antibody, showing some LEPR+ cells localizing with *Adq*-GFP+ marrow stromal cells (*arrow*). **d**, Immunolocalization of alpha-smooth muscle actin (α-SMA) and endomucin (EMCN) in the femur bone marrow of a 4-month-old *Adq-mTmG* mouse, showing an Adq-GFP+ and αSMA+ pericyte and EMCN-labeled sinusoids surrounded by *Adq-*GFP+ perivascular cells. **e**, Representative confocal images of tail vertebrae and calvaria. GFP labelling was found in tail vertebrae adipocytes (ad) and in calvaria stromal cells (*arrow*), but not in bone (*b*). **f**, Confocal microscopy analysis of different organs from *Adq-mTmG* mice showing GFP-labeling restricted to adipocytes in gonadal white adipose tissue (gWAT), inguinal subcutaneous white adipose tissue (iWAT) and brown adipose tissue (BAT). The GFP-labeled adipocytes in the Kidney panel are those of the perirenal adipose tissue.

**Figure S2.**

**
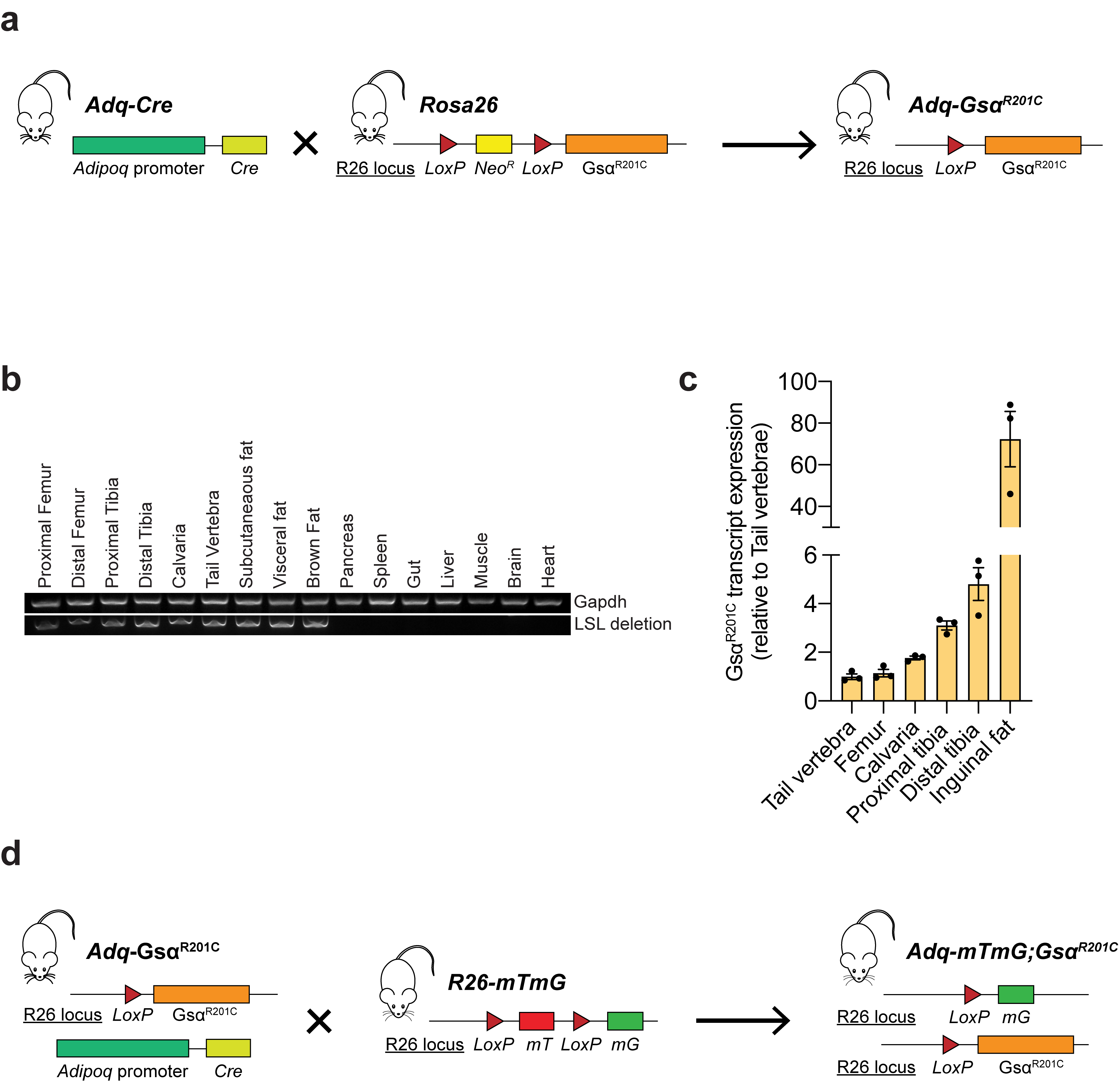
**

**Fig. S2 | a,** Experimental scheme showing the generation of *Adq*-*Gsα^R201C^* mice. **b**, Detection of the *Neo^R^* Stop cassette deletion by PCR on genomic DNA isolated from various tissues of *Adq*-*Gsα^R201C^* mice. Cre recombination occurred in skeletal segments and fat tissues but not in other organs. **c**, Gsα^R201C^ expression in different skeletal segments and in inguinal fat as assessed by qPCR. **d**, Experimental scheme showing the generation of *Adq-mTmG;Gsα^R201C^* mice.

**Figure S3.**

**
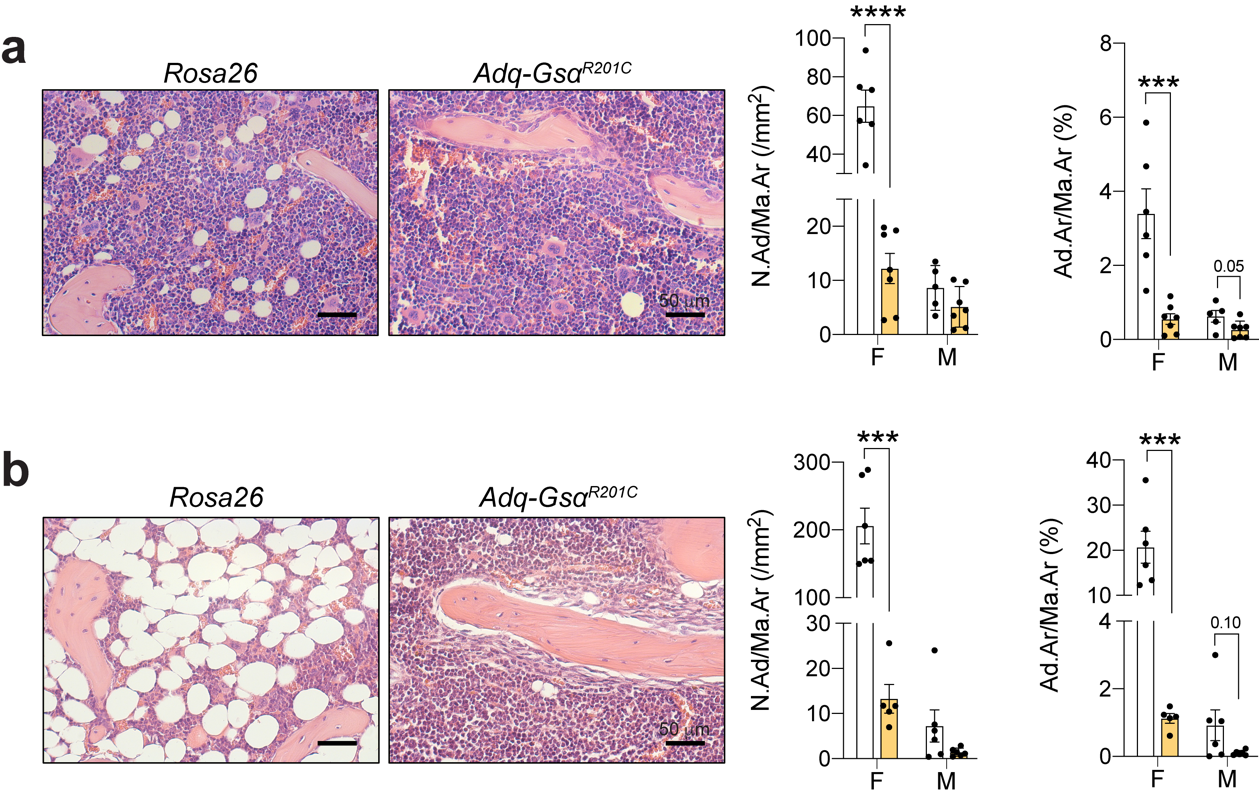
**

**Fig. S3 | a, b,** Representative histological pictures from H&E-stained sections and histomorphometric analysis of bone marrow adiposity in distal femur from 3- (**a**) and 9- (**b**) month-old mice. N.Ad/Ma.Ar: number of adipocytes per marrow area. Ad.Ar/Ma.Ar: adipocytes area per marrow area. F: females. M: males. Data are presented as mean ± SEM. Statistical analysis was performed using Student t test; ****p*<0.001, *****p*<0.0001. The exact p-value was reported on male column bars.

**Figure S4.**


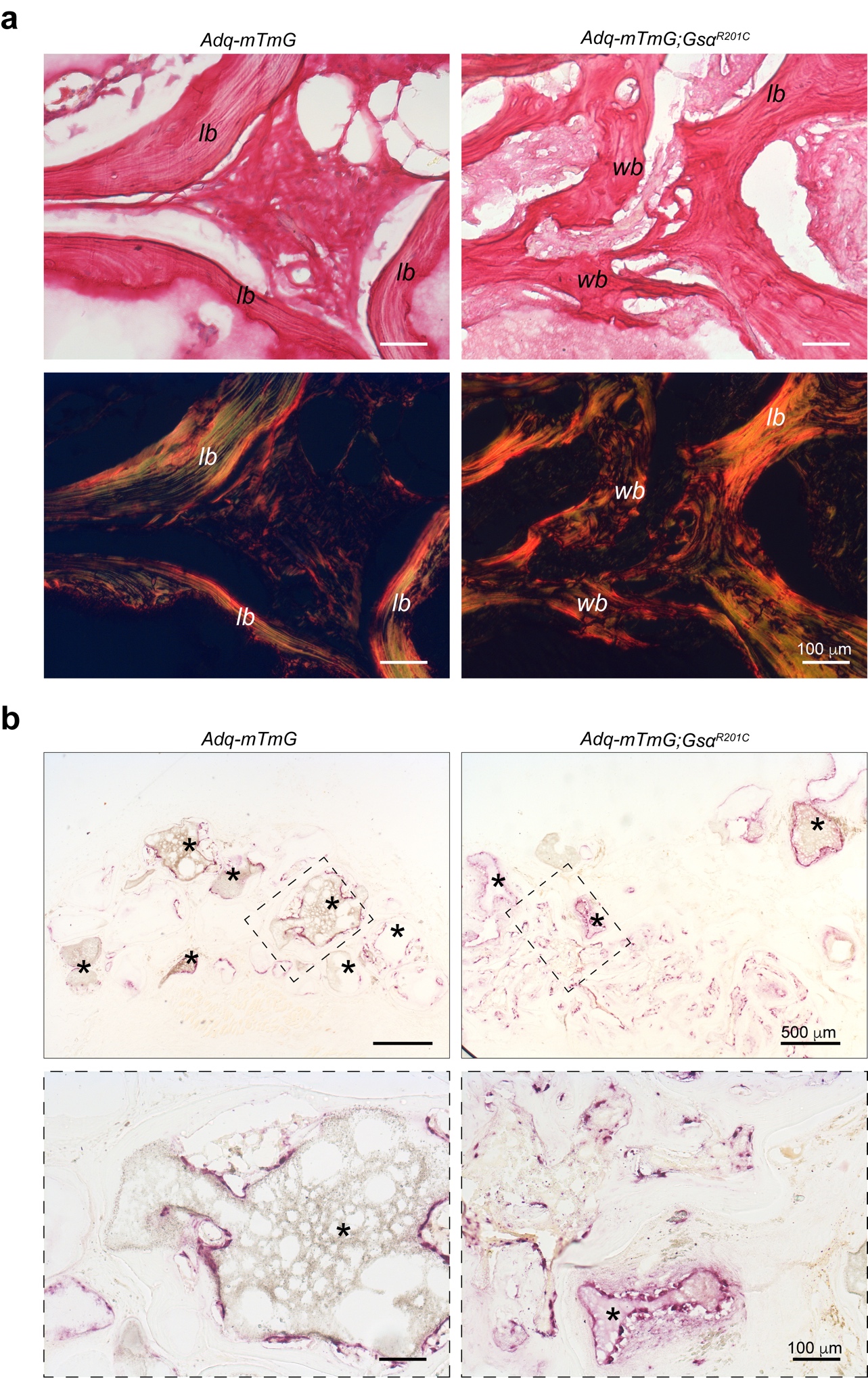


**Fig. S4 | a,** Transmitted and polarized (PL) light microscopy views of Sirius red-stained sections showing lamellar bone (*lb*) in *Adq-mTmG* control transplants and mixed lamellar and woven bone (*wb*) in *Adq-mTmG;Gsα^R201C^* transplants. **b,** TRAP-stained sections from transplants of BMSCs isolated from long bones. TRAP-stained osteoclasts (red color) are evident in both *Adq-mTmG* control and in *Adq-mTmG;Gsα^R201C^* samples in which they are more numerous. Please note that while in *Adq-mTmG* control mice osteoclasts adhere mainly to the carrier surfaces, in *Adq-mTmG;Gsα^R201C^*, in which carrier particles (*asterisk*) are markedly reduced, osteoclasts adhered also to the surfaces of the newly formed bone.

**Figure S5.**


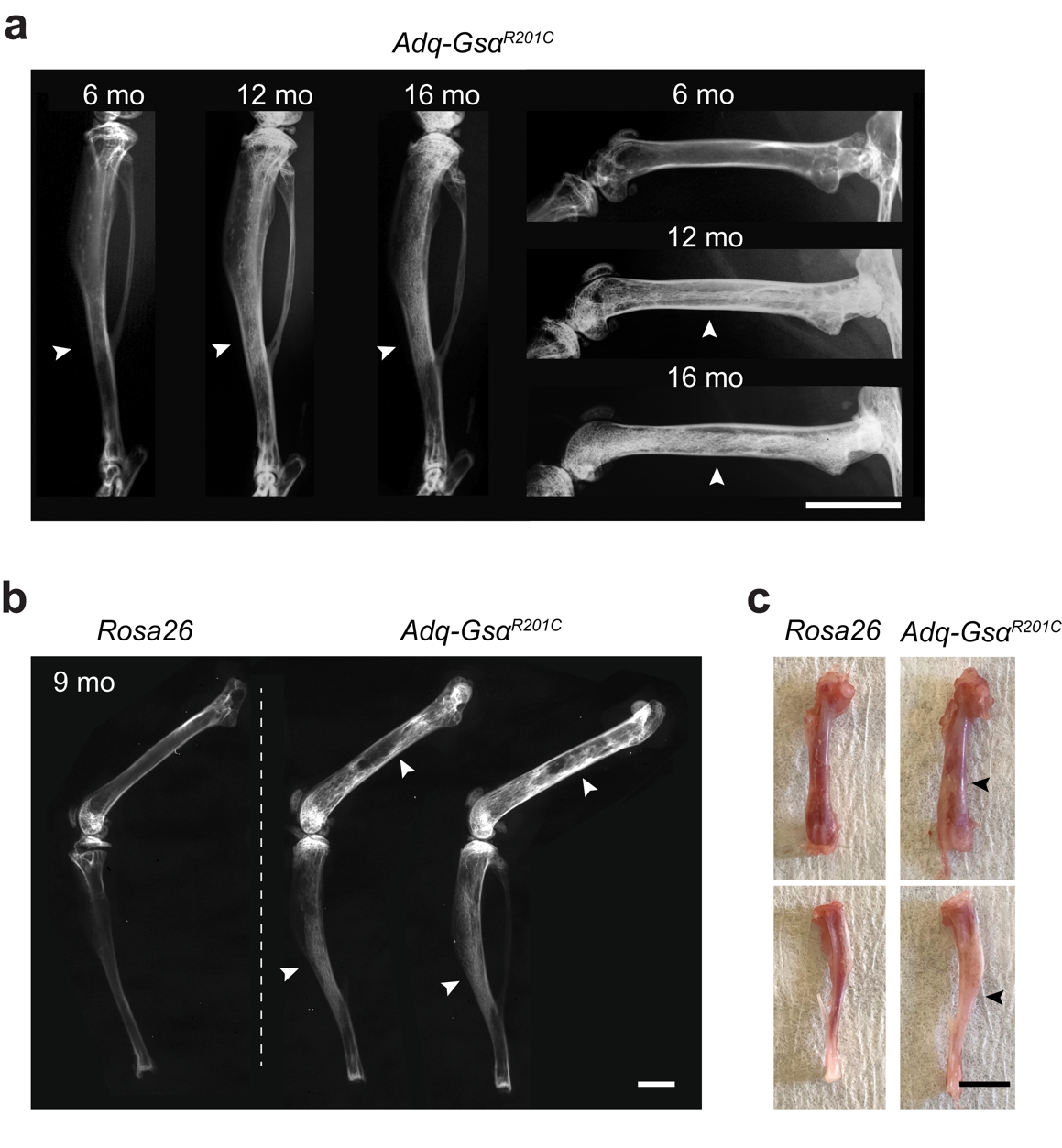


**Fig. S5** **| a,** Radiographic time course of tibiae and femurs from an *Adq-Gsα^R201C^* female mouse showing the progressive increase of intramedullary bone (*arrowhead*) in the diaphysis. Note the earlier appearance of the diaphyseal radiodensity in the tibia compared to femur. **b**, Radiographic analysis of dissected hind limbs from 9-month-old mice showing the intramedullary bone (*arrowhead).* **c**, Macroscopic appearance of femurs and tibiae from 9-month-old mice. Note the whitening of the medullary canal in *Adq-Gsα^R201C^* samples (*arrowheads*) due to bone deposition. Scale bars 5 mm.

**Figure S6.**


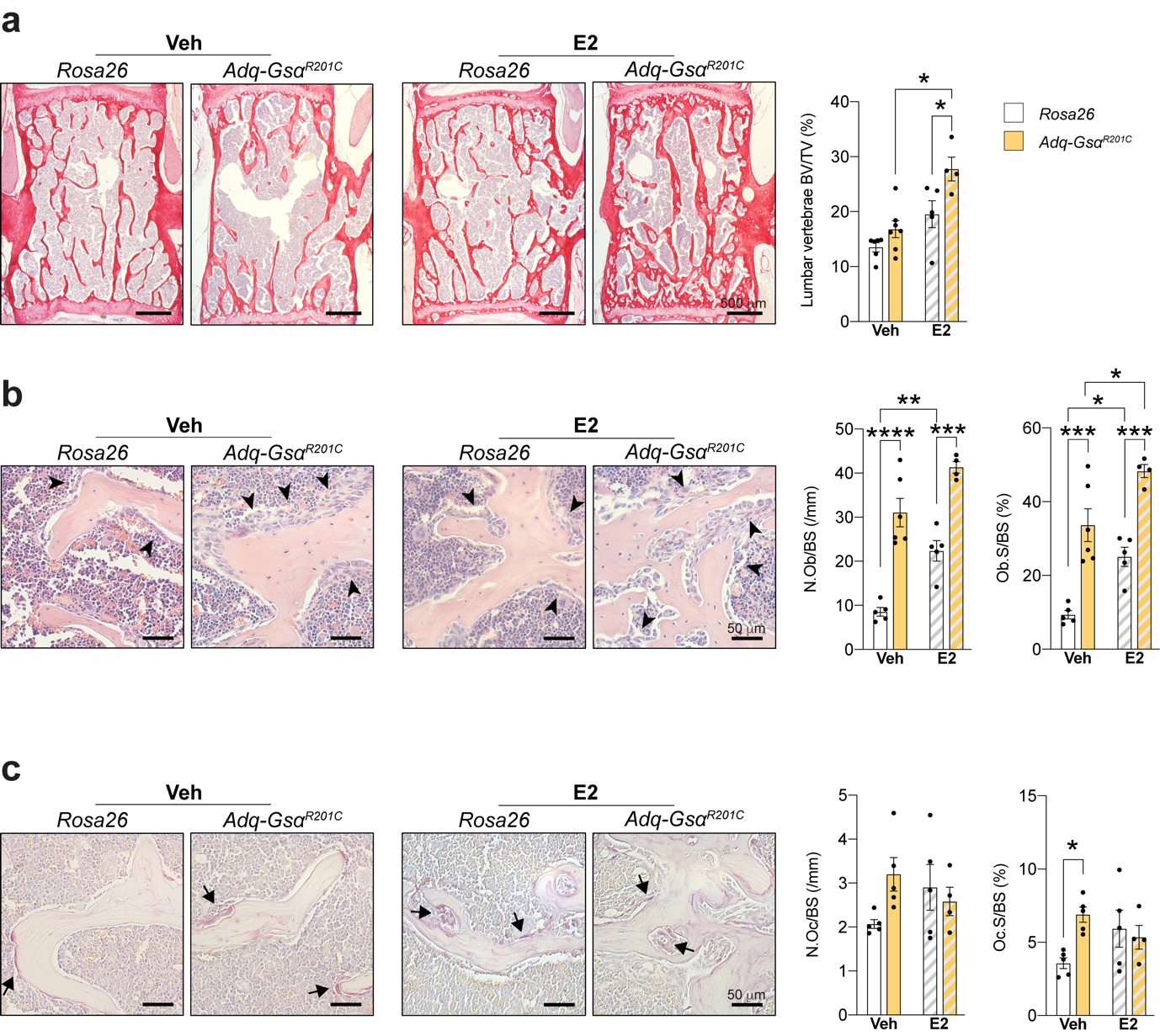


**Fig. S6** **| a,** Representative histological pictures from Sirius red-stained sections and histomorphometric analysis of trabecular bone of lumbar vertebrae after E2 treatment of male mice. BV/TV: bone volume per tissue volume. **b**, Representative images from H&E-stained sections of lumbar vertebrae from Veh- and E2-treated male mice showing osteoblasts (*arrowhead*) and quantitative histomorphometry of osteoblast parameters. N.Ob/BS: number of osteoblasts per bone surface. Ob.S/BS: osteoblast surface per bone surface. **c**, TRAP histochemistry highlighting osteoclasts (*arrow*) and quantitative histomorphometry of osteoclast parameters in trabecular bone of lumbar vertebrae after E2 treatment. N.Oc/BS: number of osteoclasts per bone surface. Oc.S/BS: osteoclast surface per bone surface.

**Figure S7.**


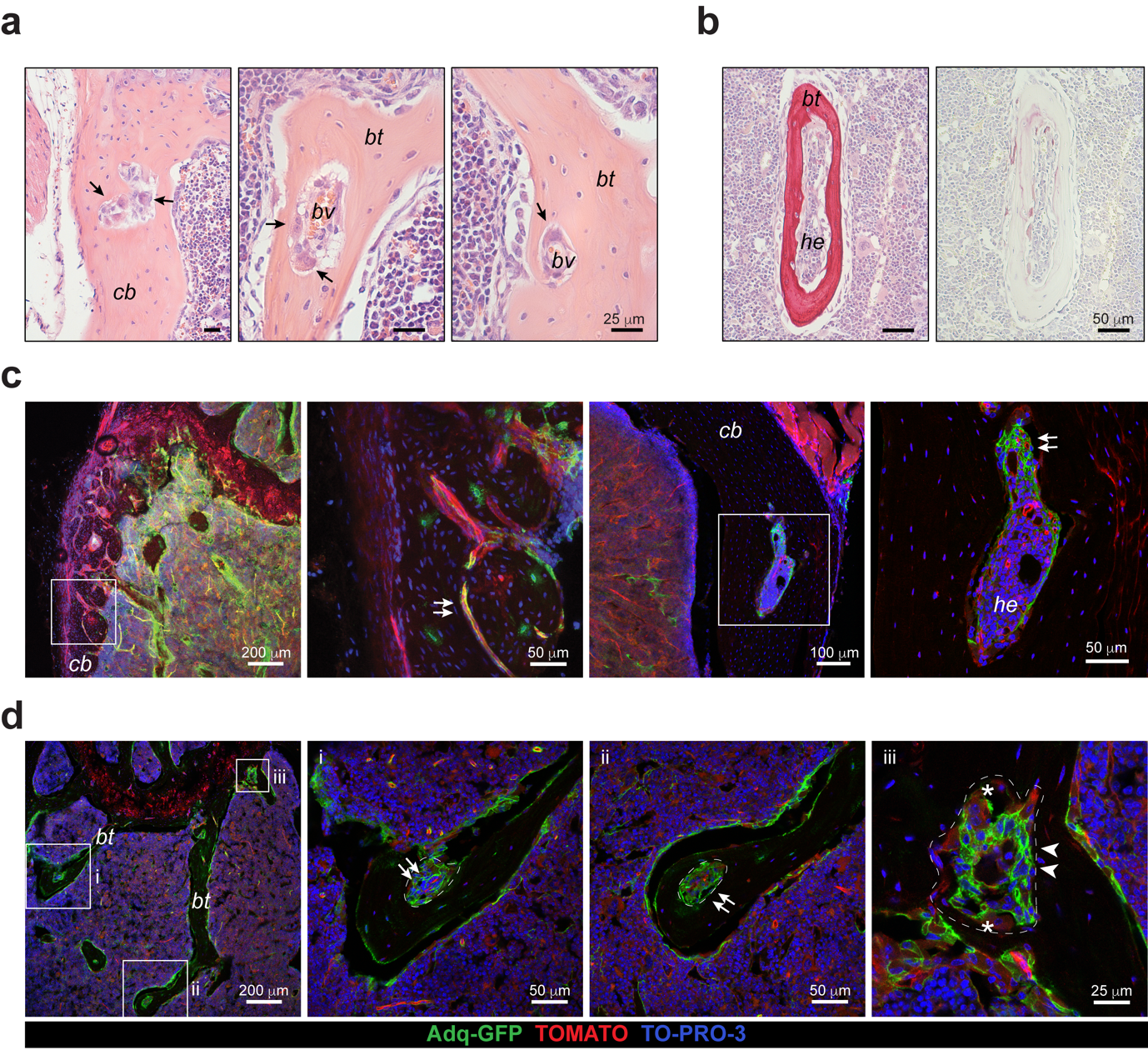


**Fig. S7 |** Intraosseous resorption in lumbar vertebrae and long bones. **a,** Intracortical lysis and trabecular tunneling resorption in lumbar vertebrae. Note the intimate connection of osteoclasts (*arrow*) with blood vessels. **b,** Sirius red and TRAP-stained sections of a lumbar bone trabecula showing tunneling resorption and localization of hematopoietic cells within the newly formed cavity. **c,** Representative confocal microscopy images of femur cortical bone from a 6-month-old *Adq-mTmG*;*Gsα^R201C^* female mouse showing GFP-expressing cells (*double arrow*) associated with blood vessels and a lytic lesion extensively refilled with hematopoietic cells. **d,** Representative confocal microscopy images of a femur from a 12-month-old *Adq-mTmG*;*Gsα^R201C^* male mouse showing tunneling resorption in bone trabeculae (*dashed line*). GFP-labeled perivascular cells (*double arrow*), as in cortical lesions, and osteoblasts (*arrowhead*). Note the presence of Tomato positive osteoclasts (asterisk). *cb*: cortical bone, *bt*: bone trabecula, *bv*: blood vessel, *he*: hematopoietic cells.
